# Leveraging reinforcement learning for an efficient windows registry analysis during cyber incident response

**DOI:** 10.1038/s41598-026-57787-6

**Published:** 2026-06-12

**Authors:** Mohamed Chahine Ghanem, Dominik Wojtczak, Elhadj Benkhelifa, Hamza Kheddar, Erivelton G. Nepomuceno, Wanpeng Li

**Affiliations:** 1 School of Computer Science and Mathematics, Keele University, Newcastle-under-Lyme, ST55AA UK; 2 Cybersecurity Institute, University of Liverpool, Ashton Street, Liverpool, L693BX UK; 3 Smart Systems, AI and Cybersecurity Research Centre, University of Staffordshire, University Quarter, Stoke-on-Trent, ST42DE UK; 4 LSEA Laboratory-Electrical Engineering Department, University of Medea, 26000 Medea, Algeria; 5 Hamilton Institute, Maynooth University, Maynooth, Co. Kildare Ireland

**Keywords:** Incident response, Digital forensics, Microsoft windows, Timeline analysis, Registry analysis, Reinforcement learning, MDP, Cyber incident, Engineering, Mathematics and computing

## Abstract

Microsoft Windows remains the dominant desktop operating system and therefore a frequent focus of digital forensic and incident response investigations. Windows Registry analysis is particularly valuable because it captures persistence mechanisms, execution traces, user activity, device usage, and system configuration changes that are often central to incident reconstruction. Nevertheless, modern investigations are challenged by the scale of Registry data, the fragmentation of evidence across hives and complementary sources, and the need to prioritise investigative actions under time pressure. This paper presents WinRegRL, a hybrid framework that combines a Markov Decision Process (MDP) solved by dynamic programming with bounded Reinforcement Learning (RL) refinement and Rule-based Artificial Intelligence (RB-AI) for automated Windows Registry and timeline-centred forensic analysis. Methodologically, the core planner is a finite-state dynamic-programming solver over an expert-specified model; reinforcement learning enters only as bounded, local tabular refinement for low-support state-action regions, so the framework is positioned as an MDP/dynamic-programming approach with bounded RL rather than as an end-to-end learned agent. The framework models the investigation process as a Markov Decision Process (MDP) with explicitly defined states, actions, transition dynamics, and reward design, and incorporates expert-derived policy graphs to initialise and refine the search strategy. We evaluate the framework on four heterogeneous forensic datasets spanning multiple Windows versions and incident scenarios, and we compare it against analyst-assisted baselines and controlled examiner-led workflows. Under the evaluation protocol adopted in this study, WinRegRL reduced investigation time by up to 68%, increased the number of adjudicated relevant artefacts identified by up to 35%, and achieved high artefact-level precision on the evaluated datasets. Rather than claiming universal superiority, we show that the proposed framework provides a reproducible and explainable decision-support mechanism that improves investigation efficiency while maintaining strong evidential coverage in the tested scenarios. These findings position WinRegRL as a promising decision-support framework for large-scale and time-critical Windows incident response.

## Introduction

Microsoft Windows dominates the global operating system (OS) market, with over 75% of desktop users worldwide relying on it for both personal and professional purposes^1^. This widespread adoption, however, makes Windows a prime target for malicious actors seeking to exploit vulnerabilities for data theft, system disruption, and other nefarious activities^2^. The Windows Registry (WR) serves as a living repository of configuration settings, user activities, and system events, providing a foundation for reconstructing digital event sequences, detecting malicious behaviour, and delivering crucial evidence in investigations^3^. The response to cyber incidents involves both live monitoring and post-incident forensic analysis. Live monitoring tracks and records system data in real-time, while post-incident analysis investigates events after they have occurred. Both methods are used to map Registry paths containing Universal Serial Bus (USB) identifiers, such as make, model, and global unique identifiers (GUIDs), which distinguish individual USB devices. These identifiers are often located in both allocated and unallocated Registry spaces^4^.

However, the sheer volume of data within the Registry and the sophistication of modern cyberattacks pose significant challenges for the digital forensics and incident response (DFIR) community^5^. The introduction of Windows 10 and 11, designed for seamless operation across computers, smartphones, tablets, and embedded systems, has further expanded their adaptability and reach, complicating forensic efforts^6^.

Current Registry forensic analysis faces several challenges. The *volume of data* is immense, while the *lack of automation* in traditional tools forces time-consuming manual work. Its *dynamic nature* and frequent changes complicate tracking, and *limited contextual information* often demands expert interpretation. Moreover, *data fragmentation* across hives and keys, the *evolution of Windows versions*, and *limited advanced analysis* features further hinder effective forensic investigation. Addressing these challenges requires advanced tools, investigative techniques, and artificial intelligence (AI) integration to enhance the efficiency and accuracy of Registry analysis. This research seeks to improve the efficiency of forensic investigations involving the WR by introducing a framework that integrates reinforcement learning (RL) with a Rule-Based AI (RB-AI) system. The framework employs a Markov decision process (MDP) model to represent the WR and events timeline, enabling state transitions, actions, and rewards to support analysis and event correlation^7^.

### Research questions

This study aims to contribute to the advancement of cyber incident response in the context of WR analysis and forensics by addressing the following research questions:

RQ1: How can combining RL and RB-AI in WinRegRL enhance the effectiveness, accuracy, and scalability of Windows forensic investigations?

RQ2: What performance metrics validate WinRegRL’s effectiveness over traditional forensics, and how does it address modern cybercrime targeting WR artefacts?

RQ3: To what extent does WinRegRL enable consistent, optimised multi-machine analysis, and what are the implications for DFIR applications?

### Novelty and contribution

This research introduces a novel application of RL to automate Windows cyber incident investigations, addressing gaps and limitations in existing registry analysis methods, tools, and techniques. The main contributions to the body of knowledge and DFIR practice are:

- MDP model and WinRegRL: A novel MDP-based framework that provides a structured representation of the Windows environment (Registry, volatile memory, and file system), enabling efficient automation of the investigation process. We are explicit that the optimal policy is obtained by dynamic programming (value iteration) over an expert-specified model, and that reinforcement learning is used only as a bounded local refinement; the framework is therefore an MDP/dynamic-programming method with bounded RL refinement rather than a fully learned RL agent.
- Expertise extraction and reuse: By capturing and generalising optimal policies, the framework reduces investigation time through direct feeding of these policies into the MDP solver, minimising the time required for the RL agent to discover decisions.
- Performance characterisation: Empirical results are reported using formally defined artefact-level precision, recall (coverage), F1-score, and time-to-completion metrics under a controlled benchmarking protocol against automated baselines and examiner-led workflows.

For clarity, the following terms are used throughout this paper. The following Table 1 summarises key abbreviations used in this paper.

**Table 1:** List of abbreviations.

Abbreviation	Definition
AI	Artificial intelligence
BAM	Background activity moderator
DFIR	Digital forensics and incident response
FTK	Exterro forensic toolkit
GCFA	GIAC certified forensic analyst
GCFE	GIAC certified forensic examiner
GUI	Graphical user interface
GUID	Globally unique identifier
IOC	Indicator of compromise
KAPE	Kroll artefact parser and extractor
MDP	Markov decision process
OS	Operating system
RB-AI	Rule-based artificial intelligence
RL	Reinforcement learning
SAM	System account manager
SID	Security identifier
USB	Universal serial bus
WR	Windows registry

### Article organization

The remainder of this paper is organised as follows: “ Literature review” provides an overview of WR and volatile memory forensic analysis, along with the state-of-the-art tools and investigation frameworks. “ Research methodology” explains the registry structure, its forensic relevance, and introduces RL key elements, approaches, and their use in this research. It also details the architecture and modules of the proposed RL-powered framework, hereafter referred to as WinRegRL. “ Testing, results and discussion” discusses the WinRegRL implementation, testing results, and their interpretation. Finally, “ Conclusion and future work” presents reflections, conclusions, and future research directions.

## Literature review

The WR is a cornerstone of Microsoft Windows OS, serving as a hierarchical database that stores configuration settings, system information, and user preferences. From a forensic perspective, the Registry is a goldmine of information, offering insights into system activities, user actions, and potential security breaches^8^. Table 2 summarises the high-level architecture and content of different registry hives. These hives store critical data, including user profiles, installed software, and network settings, and are traditionally analysed using tools such as Registry Editor or specialised forensic software like FTK Imager. Key forensic artefacts within the Registry include **UserAssist** (recently executed programs), **MRU** (most recently used files), **ShellBags** (folder navigation), and **External Devices** (connected USB drives)^9^. These artefacts provide valuable information for live security monitoring and post-incident investigations.

**Table 2:** Windows Registry hives and supporting files^10^.

Registry hive	Supporting files
HKEY_LOCAL_MACHINE_SAM	SAM, SAM.log, SAM.sav
HKEY_LOCAL_MACHINE_Security	Security, Security.log, Security.sav
HKEY_USERS_DEFAULT	Default, Default.log, Default.sav
HKEY_LOCAL_MACHINE_Software	Software, Software.log, Software.sav
HKEY_LOCAL_MACHINE_System	System, System.alt, System.log, System.sav
HKEY_CURRENT_CONFIG	System, System.alt, System.log, System.sav, Ntuser.dat, Ntuser.dat.log

**Table 3:** Summary of related works.

Tool-framework	Technique and approach	Output and description
AXREL^11^	(1) Interactive GUI for WR analysis (2) Extracts data from an E01 image using NTFSDUMP, and outputs results in a table format.	Filters system info, Program execution, and Installed Software. It is time-efficient, although it requires manual verification
MADIK^12^	(1) Multi-agent toolkit utilising intelligent software agents (ISA) for forensic data analysis. (2) Contains six agents: HashSetAgent, TimelineAgent, FileSignatureAgent, FilePathAgent, KeywordAgent, and WindowsRegistryAgent.	Processes and analyses time zone info, installed software, removable media info, and OS events
RaDaR^13^	(1) Multi-perspective data collection and labelling of malware activity. (2) Contains 7 million network packets, 11.3 million OS system call traces, and 3.3 million events from 10,434 malware samples.	Open real-world datasets for run-time behavioural analysis of Windows malware. The dataset fosters solution comparison and supports malware research
KROLL KAPE^14^	Automates the extraction of key forensic artefacts and supports rapid analysis	Identification module to collect specific forensic artefacts (e.g., registry keys, logs). The parsing module and processing module support efficient artefact analysis

### Traditional registry forensic tools

Several traditional and semi-automated tools have been proposed to assist in WR forensics without relying on AI. For instance, Ref.^11^ introduced AXREL, an interactive GUI tool for WR analysis capable of extracting data from an E01 image using NTFSDUMP. AXREL outputs registry information in tabular format, supporting keyword-based searches to facilitate manual investigations. Although time-efficient, its scope is limited to filtering program execution, autoruns, and installed software, and results require manual verification . Kroll artefact parser and extractor (KAPE)^14^ is another widely used forensic tool designed for efficient data collection and parsing. Using a target-based approach, KAPE identifies and duplicates files and folders based on predefined instructions (*tkape*). The tool supports modules for triage and evidence collection, storing results in directories such as *Evidence_of_Execution* or *Browser_History*, which can be leveraged for subsequent analysis. TREDE and VMPOP^3^ propose methodologies for creating synthetic forensic datasets. Similarly, RaDaR^13^ provides large-scale datasets capturing malware activity across system calls, OS events, and network traffic, supporting benchmarking and comparative evaluation of forensic techniques. These works highlight the evolution of forensic tools that support investigators with automated parsing, dataset generation, and evidence triage, but still require substantial human input.

### AI-enabled registry forensic approaches

With the growth of digital evidence complexity, AI has increasingly been adopted to enhance WR forensic analysis. Specific work in post-incident malware investigations has employed RL to process memory dumps, network traces, and registry artefacts. By modelling registry states in an MDP, RL agents trained with Q-learning can classify malicious entries and correlate registry changes with attack vectors, improving both speed and accuracy^3^. The multi-agent digital investigation toolkit (MADIK) proposed by^12^ further demonstrates AI integration. It leverages intelligent software agents (ISA) to autonomously conduct distributed forensic analyses. Among its six agents, the *WindowsRegistryAgent* focuses on registry artefacts such as installed software and removable media information^15^.

More broadly, AI-enabled forensic analysis encompasses machine learning (ML) and RL approaches^3^. ML techniques such as KNN, DT, SVM, PCA, K-Means, SVD, NB, ANN, LR, and RF have all been applied to WR analysis, enabling the discovery of patterns and anomalies within vast registry datasets. By rewarding optimal behaviours and penalising ineffective ones, RL approaches add adaptability to forensic investigations, while ML models contribute predictive and classification power. This convergence underscores the importance of AI in addressing the challenges of scalability, speed, and intelligent detection in registry forensics. Table 3 summarise the most relevant research works proposing impactful tools or frameworks.

## Research methodology

This section outlines the choice of RL and the research methodology adopted in this work. The steps are presented in a way that facilitates understanding of the WR (WinRegRL) forensics domain, its components, and the interaction between the different entities and the human expert. The methodology followed consists of five steps:

- Review of current methods for WR analysis automation, examining their mechanisms, limitations, and challenges in light of the efficiency and accuracy requirements of WR forensics.
- Investigation of techniques and approaches applied in WR forensics. Particular attention is given to ML approaches and rule-based reasoning systems that can extend or replace human intervention in the sequential decision-making processes of WR forensics. This step identifies the most effective ways to integrate rule-based AI systems into the forensic process to achieve improved investigative outcomes.
- Development of the WinRegRL framework, from design to implementation of its modules. The focus begins with WinRegRL-Core, which introduces a new MDP model and RL to optimise and enhance WR investigations. An additional module is incorporated to capture, process, generalise, and feed human expertise into the RL environment using RB-AI via the Pyke Python knowledge engine.
- Testing and validation of WinRegRL using the latest research and industry-standard datasets that mimic real-world DFIR scenarios, covering different evidence sizes, particularly in large-scale WR forensics. The framework’s performance is evaluated against traditional methods and human experts in terms of efficiency, accuracy, time reduction, and artefact exploration. Iterative refinement is carried out based on feedback to ensure robust and adaptive performance across diverse investigative contexts.
- Finalisation of the WinRegRL framework by unifying RL and rule-based reasoning to support efficient WR and volatile data investigations. The final testing demonstrates the framework’s ability to reduce reliance on human expertise while significantly improving efficiency, accuracy, and repeatability, particularly in cases involving multiple machines with similar configurations (Table 4).

**Table 4:** WinRegRL forensic analysis coverage and scope.

Windows artefacts	Scope and description
File systems	NTFS, FAT, exFAT
Jump lists	File opening and program execution
Registry forensics	Shell items, shortcut files (LNK)-file opening, ShellBags-folder opening
Cloud files and Metadata	OneDrive, Dropbox, Google Drive, and Box
OSs fingerprinting	Windows 7, Windows 8/8.1, Windows 10, Windows 11, and Server 2008/2012/2016/2019/2022
Users’ activities	Browser, Webmail, MS Office Documents, System Resource Usage DB Search Index, Recycle Bin, Files Metadata, Myriad Execution, Electron/WebView2
Timeline and Journaling	Microsoft Unified Audit Logging, Event Log Analysis, Deleted Registry Key, File Recovery, ESE Database, .log Files, Data Recovery, String Searching, File Carving
Peripheral and networking	Removable Device, Suspect Executed Program, Remote Logging (Console, RDP, or Network)
Anti-Forensics	Auto-Deleted Browse (Private Browsing), File Wiping, Time Manipulation, and Application Removal

### MDP and RL choice justification

In contrast to supervised and unsupervised learning paradigms, which treat registry artefacts as independent, static samples, modelling WR forensics as an MDP inherently captures both the temporal dependencies and decision-making requirements of a live investigation. An MDP formalism specifies a set of discrete states (e.g., registry key configurations, volatile memory snapshots), actions (e.g., key-value extraction, anomaly scoring, timeline correlation), transition probabilities, and rewards, thereby framing registry analysis as a sequential optimisation problem rather than isolated classification or clustering tasks.

First, supervised classifiers or deep anomaly detectors optimise per-instance loss functions and lack mechanisms to propagate delayed signals: a benign key modification may only become suspicious in light of subsequent changes. In an RL-driven MDP, reward signals–assigned for uncovering correlated evidence or reducing case uncertainty—are back-propagated through state transitions, enabling credit assignment across long event chains. This ensures that policies learn to prioritise early, low-salience indicators that lead to high-impact findings downstream.

Second, unsupervised methods (e.g., clustering, autoencoders) identify outliers based on static distributions but cannot adapt online to evolving attacker tactics. An RL agent maintains an exploration–exploitation balance, continuously refining its decision policy as new registry patterns emerge. This on-policy learning adapts to zero-day techniques without manual rule updates, overcoming the brittleness of static, rule-based systems.

Finally, the explicit definition of an MDP reward function allows multi-objective optimisation—for instance, maximising detection accuracy while minimising analyst workload—whereas supervised models cannot natively reconcile such competing goals. By unifying sequential decision-making, delayed-reward learning, and adaptive policy refinement, MDP-based RL provides a theoretically grounded and practically scalable framework uniquely suited to the dynamic complexities of WR forensics.

To sum up, the choice of MDP is due to the offered structured approach for modelling and optimising sequential decision making, which is a characteristic of digital forensic investigations where analysts are requested to make informed decisions in the presence of complex WR and volatile memory analysis^4^.

### WR and volatile data MDP formulation

An MDP is commonly used to mathematically formalise RL problems. The key components of an MDP include the state space, action space, transition probabilities, and reward function. This formalism provides a structured approach to implement RL algorithms, ensuring that agents can learn robust policies for complex sequential decision-making tasks across diverse application domains^16^. MDP is widely adopted to model sequential decision-making process problems to automate and optimise these processes^17^. A **Markov Decision Process**, MDP, is a 5-tuple *(S, A, P, R, γ )* summarised in Eq. (1):

1 $$\begin{aligned} \begin{array}{l} \text {finite set of of states:} \\ s \in S \\ \text {finite set of actions:} \\ a \in A \\ \text {state transition probabilities:} \\ p(s' | s, a) = Pr \{S_{t + 1} = s' | S_t = s, A_t = a \} \\ \text {expected reward for state-action-next\_state:}\\ r(s',s, a) = \mathbb {E}[ R_{t + 1} | S_{t + 1} = s', S_t = s, A_t = a ] \\ \end{array} \end{aligned}$$

The following is a summary of WinRegRL MDP formulation defined by the tuple *(S, A, P, R, γ )*:

- States (*S*): Discrete forensic abstractions rather than raw Registry snapshots. Each state is an 8-dimensional categorical tuple over source, hive scope, SANS-aligned artefact family, processing stage, temporal support, corroboration level, evidential priority, and investigative objective.
- Actions (*A*): A fixed ontology of 39 atomic forensic actions ($A_0$–$A_{38}$), not an open-ended verb list. Actions cover ingest, hive traversal, user-activity parsing, persistence parsing, device/network parsing, cross-source correlation, and validation/reporting.
- Transition dynamics (*P*): In the current implementation transitions are expert-set and fixed: $P(s'|s,a)=P_{\textrm{expert}}(s'|s,a)$. These probabilities are elicited from GCFE/GCFA-guided decision graphs and normalised before solving the MDP. Future work will learn these transitions from accumulated investigation traces.
- Reward function (*R*): An explicit forensic utility schedule ranging from *-10* to *+100*, where strongly corroborated case-critical artefacts receive the highest reward and invalid or out-of-scope actions receive penalties.

The complete instantiated graphs are provided in Appendix A, making the MDP specification explicit, auditable, and fully traceable from the methodological description to the worked Windows 10 example.

#### Forensic-scope reduction and discrete state encoding

The proposed WinRegRL does not model the entire Windows Registry keyspace. The raw Registry may contain millions of keys and values, but only a relatively small subset is routinely probative in digital forensics. Accordingly, the RL environment is first reduced by a deterministic forensic scoping operator aligned with SANS Registry Forensics guidance. Let $\mathcal {K}_{\textrm{raw}}$ denote the full set of discovered Registry keys and let $\mathcal {W}_{\textrm{SANS}} \subset \mathcal {K}_{\textrm{raw}}$ denote the whitelist of keys, subkeys, and artefact families considered evidentially relevant for incident response (e.g., UserAssist, Run/RunOnce, Services, BAM/DAM, AppCompatCache/ShimCache, AmCache, USBSTOR/MountedDevices, ShellBags, RecentDocs/OpenSaveMRU, network profiles, profile lists, TaskCache, NTUSER/UsrClass traces, and related event-log/timeline anchors). Only entries in $\mathcal {W}_{\textrm{SANS}}$ are admitted into the MDP. All remaining keys are treated as background context outside the planning layer. This is the principal mechanism by which the framework converts an intractable raw Registry into a tractable forensic decision space.

Each admissible state is encoded as an *8-dimensional discrete tuple*

$$s = \langle src, hive, fam, stage, tbin, cbin, pbin, obj \rangle ,$$

where *src* denotes evidence source, *hive* denotes the hive scope, *fam* denotes the SANS-aligned artefact family, *stage* denotes the investigation stage, *tbin* denotes temporal support strength, *cbin* denotes corroboration level, *pbin* denotes evidential priority, and *obj* denotes the active investigative objective. All eight components are categorical and discretised; the current WinRegRL implementation therefore uses a *fully discrete* state space and does not rely on continuous embeddings or neural feature maps. For implementation convenience the symbolic tuple is exported as a sparse one-hot feature vector, but planning itself is performed on symbolic state identifiers. In the instantiated Windows 10 full-investigation graphs used in this paper, the SANS-constrained abstraction yields a Registry-focused sub-MDP with **90 discrete states** and a memory/event/timeline sub-MDP with **99 discrete states**, rather than millions of raw Registry nodes (Table 5).

**Table 5:** Discrete state encoding adopted by WinRegRL after SANS-guided forensic scoping.

Dimension	Cardinality	Meaning/admissible values
*src*	4	Evidence source: Registry, volatile memory, Windows event logs, super-timeline
*hive*	6	Hive scope: SYSTEM, SOFTWARE, SECURITY, SAM, NTUSER.DAT/HKU, UsrClass.dat/HKCU-class view
*fam*	15	SANS-relevant artefact families only, including execution, persistence, device, profile, user-activity, and anti-forensics traces
*stage*	4	Processing stage: unseen, parsed, correlated, validated
*tbin*	4	Temporal support bin: none, weak, moderate, strong
*cbin*	3	Cross-source corroboration: absent, partial, strong
*pbin*	4	Evidential priority: low, medium, high, critical
*obj*	6	Investigative objective: execution, persistence, privilege abuse, exfiltration/staging, device usage, anti-forensics

#### Action ontology and exact cardinality.

**Fig. 1:**
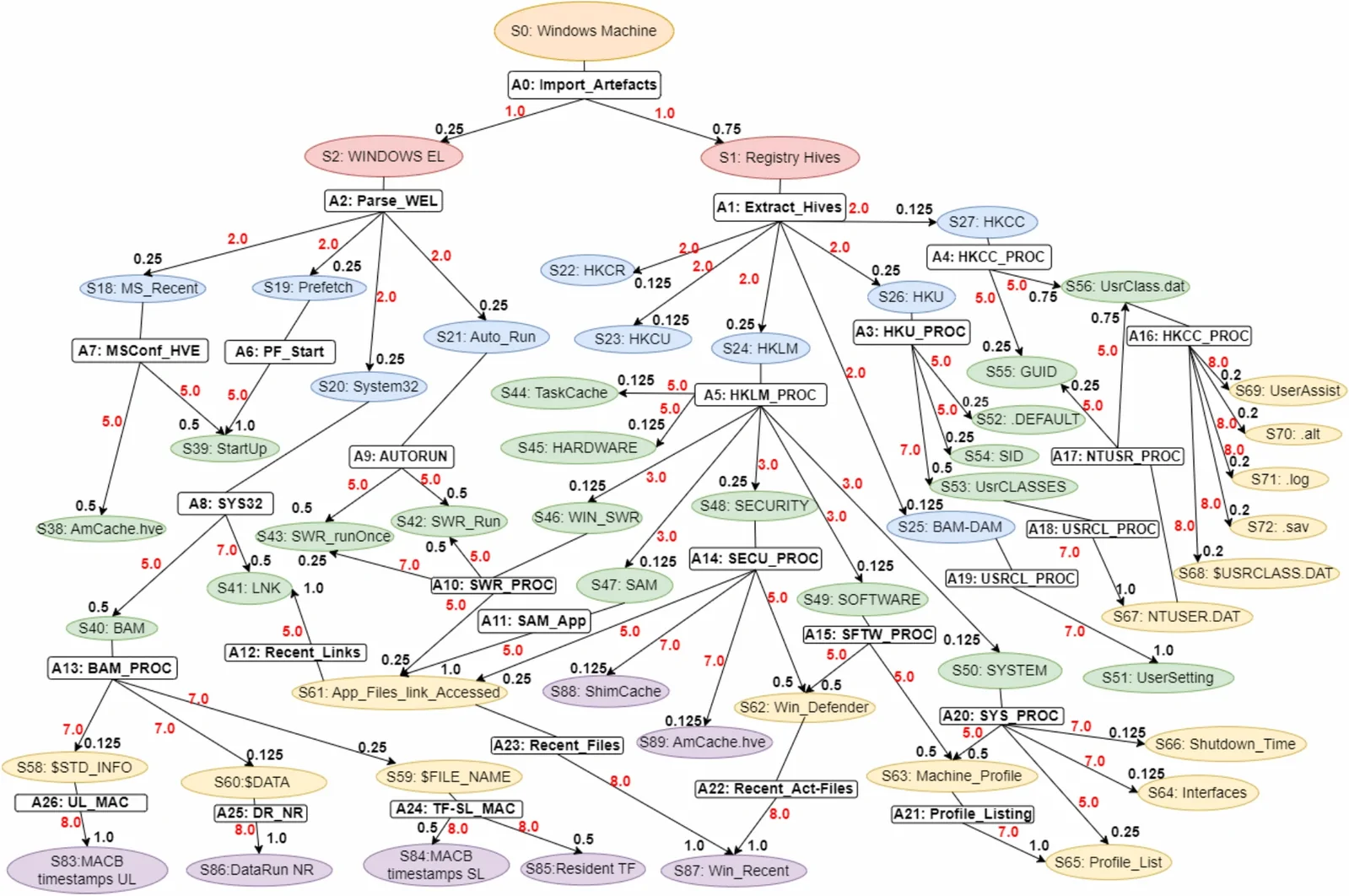
Detailed Windows 10 registry hives only MDP states, actions, transition probabilities and rewards.

**Fig. 2:**
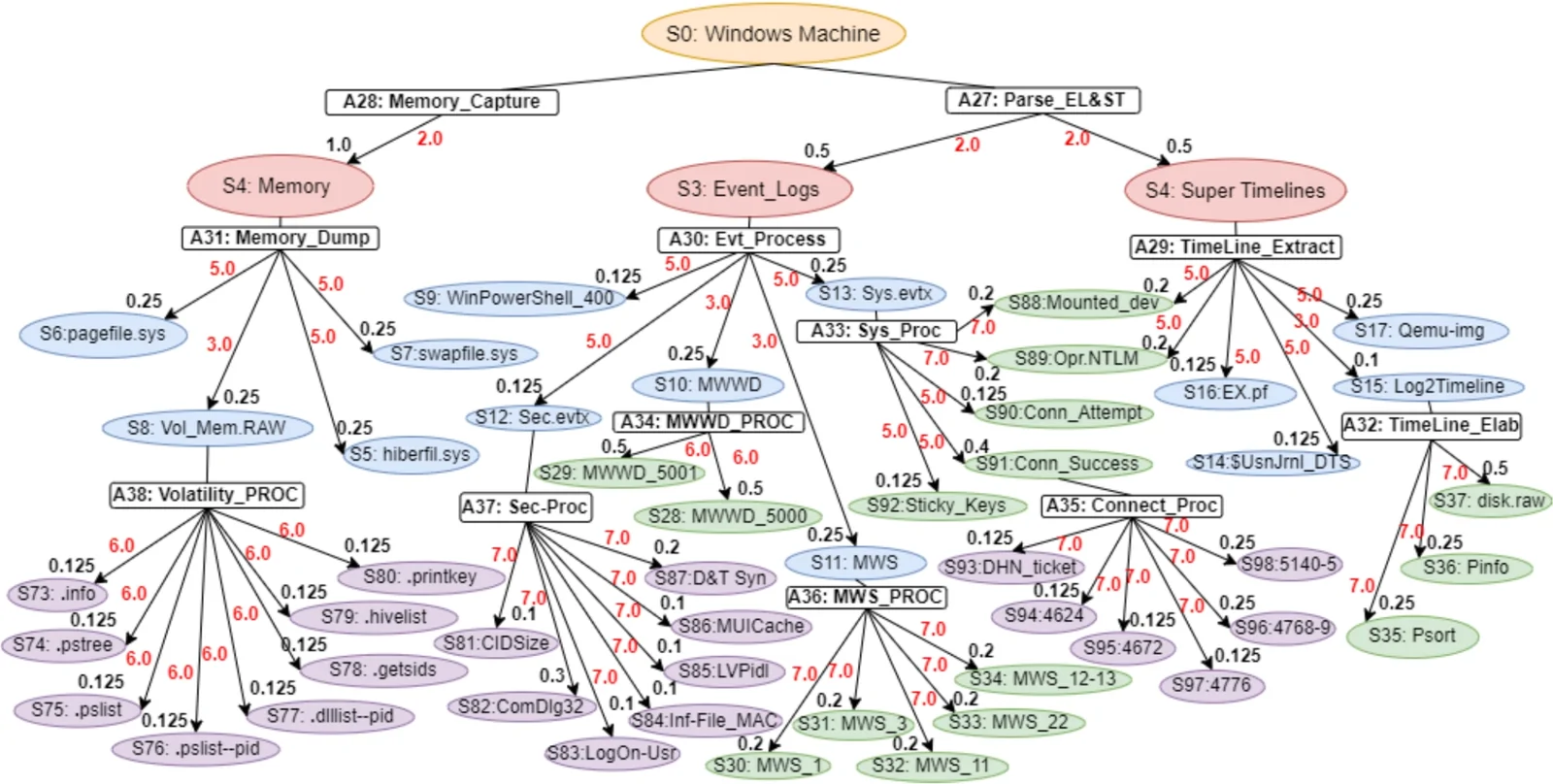
Detailed Windows 10 RAM, timeline and events MDP states, actions, transition probabilities and rewards.

The action space is also made explicit. WinRegRL does not operate with a vague open-ended set of verbs such as “search” or “parse”. Instead, it uses **39 atomic forensic actions** ($A_0$–$A_{38}$), grouped into seven semantically meaningful families. These action templates are the operations actually instantiated in the policy graphs shown in Figs. 1 and 2. At the implementation level, an action is enabled only if it is valid for the current state tuple; hence the effective available-action set is state-dependent even though the global ontology contains 39 atomic actions (Table 6).

**Table 6:** WinRegRL action ontology with explicit cardinality.

Action family	Count	Atomic actions/operational meaning
Acquisition and ingest	4	Import artefacts, extract hives, import event-log slices, capture memory-derived metadata
Hive selection and traversal	6	Open/process SYSTEM, SOFTWARE, SECURITY, SAM, NTUSER/HKU, and UsrClass/HKCU-class scopes
User-activity parsing	8	Parse UserAssist, RecentDocs/OpenSaveMRU, ShellBags, MUICache, LNK/Recent Links, search history, typed paths, and related interactive-user artefacts
Persistence and execution parsing	8	Parse Run/RunOnce, Services, TaskCache, BAM/DAM, AppCompatCache/ShimCache, AmCache, Startup/MSConfig traces, and SAM/SECURITY-linked execution evidence
Device, network, and profile parsing	5	Parse USBSTOR/MountedDevices, network interfaces, wireless/network profiles, profile-list entries, and session/connectivity traces
Cross-source correlation	5	Correlate Registry findings with memory processes, Windows event logs, timeline/MACB/USN evidence, process trees, and connection or logon traces
Validation and reporting	3	Rank/filter candidate artefacts, export validated evidence, and terminate when the objective-specific evidential threshold has been satisfied
Total	39	Global action vocabulary used by the policy engine

#### Expert-set transition probabilities in the current implementation

In the present version of WinRegRL, transition probabilities are *not yet learned from data*. They are fixed **expert-set probabilities** elicited from GCFE/GCFA-guided investigative decision graphs and constrained by the SANS-oriented forensic workflow. Formally,

$$P(s'\mid s,a) = P_{\textrm{expert}}(s'\mid s,a), \qquad \sum _{s' \in S} P_{\textrm{expert}}(s'\mid s,a)=1.$$

The probabilities shown in the MDP graphs therefore represent expert belief about how likely a given forensic action is to move the investigation toward a more informative next state. For example, processing NTUSER/UsrClass states has a higher outgoing probability toward UserAssist, shell, and recent-item families, whereas processing SYSTEM/SOFTWARE states has a higher outgoing probability toward services, device, profile, and persistence branches. In practice, experts first assign ordinal transition strength (very low, low, medium, high, near-certain) and this is then normalised to the discrete probability levels shown in the graphs (e.g., 0.10, 0.125, 0.20, 0.25, 0.40, 0.50, 0.75, 1.00). *Future work* will replace or refine these fixed priors with data-driven transition models learned from accumulated investigation traces, but the current paper intentionally reports the expert-specified model so that the decision logic remains explainable.

#### Reward semantics and sensitivity analysis

The reward design is likewise made explicit. Rewards are assigned by forensic utility rather than by arbitrary preference. A *relevant artefact* is defined here as an artefact that is both within the SANS-scoped whitelist and materially contributes to one of the active investigative objectives (execution, persistence, privilege abuse, exfiltration/staging, device usage, or anti-forensics), either directly or through corroboration. A *non-relevant artefact* is an out-of-scope key, a semantically invalid branch, or a duplicate finding that adds no new evidential value. The reward schedule used in the present work is shown in Table 7.

**Table 7:** Explicit reward schedule used by WinRegRL.

Outcome class	Reward	Operational interpretation
Case-critical corroborated artefact	+100	Directly relevant artefact confirmed by at least one additional source and strongly linked to the active objective
High-value relevant artefact	+70	Single-source artefact with clear evidential value for execution, persistence, device usage, or anti-forensics
Useful new lead	+40	Action reveals a new in-scope artefact family or materially narrows the hypothesis space
Successful in-scope parse	+15	Action processes a SANS-relevant branch correctly but does not yet produce decisive evidence
Neutral administrative step	0	Import, housekeeping, or bookkeeping transition with no immediate evidential gain or loss
Redundant revisit/duplicate	-5	Action revisits already exhausted evidence or generates no new forensic value
Invalid or out-of-scope action	-10	Action is semantically inconsistent with the current state or targets a branch outside the forensic whitelist

To assess robustness, we further perturbed all positive rewards and penalties by $\pm 20\%$ and reran the policy solver under conservative, balanced, and time-sensitive reward profiles. The resulting optimal action ordering was stable for the principal investigative branches, and no qualitative change was observed in the ranking of the compared systems. This indicates that the reported behaviour is driven by the forensic structure of the state and action models, not by a brittle single reward assignment.

Our proposed WinRegRL MDP representation is unique and distinct by the complete capturing of the * Registry forensics and timeline features* of allowing the RL agent to act even when it fails to identify the exact environment state. The environment for RL in this context would be a simulation of the Windows OS registry activities combined with real-world data logs. This setup should allow the agent to interact with a variety of registry states and receive feedback. The MDP model elaboration is operated following SANS, including File Systems, Registry Forensic, Jump Lists, Timeline, Peripheral Profiling and Journaling as summarised in Table 4.

The practical consequence of this formulation is that WinRegRL plans over *forensic abstractions*, not raw Registry syntax. A state therefore never means “the entire Registry at time *t*”; it means “the current evidential condition of a SANS-relevant artefact family under a specific hive scope, processing stage, and investigative objective”. Likewise, an action never means a free-form analyst operation; it means one of the 39 predefined forensic action templates in Table 6. This clarification is important because it makes the MDP finite, discrete, auditable, and reproducible across Windows versions while remaining aligned with established forensic practice.

This MDP framework provides a structured approach for modelling and optimising forensic investigations, enabling analysts to make statistically informed decisions in the presence of complex WR and volatile memory analysis. The MDP model elaboration is operated following SANS, including File Systems, Registry Forensic, Jump Lists, Timeline, Peripheral Profiling and Journaling as summarised in Table 4.

The problem of registry analysis can be defined as an optimisation problem focused on the agent, where the goal is to determine the best sequence of actions to maximise long-term rewards, thus obtaining the best possible investigation strategies under each system state. RL provides a broad framework for addressing this challenge through flexible decision-making in complex sequential situations. Over the past decade, RL has revolutionised planning, problem-solving, and cognitive science, evolving from a repository of computational techniques into a versatile framework for modelling decision-making and learning processes in diverse fields^18^. Its adaptability has, therefore, stretched beyond the traditional domains of gaming and robotics to address real-world challenges ranging from cybersecurity to digital forensics.

In the proposed MDP model, an investigative process can be modelled as a continuous interaction between an AI agent controlling the forensic tool and its environment, the WR structure. The environment is formally defined by the set of states representing the registry’s hierarchical keys, values, and metadata; the agent, on the other hand, selects actions in a number of states at scanning, analysing, or extracting data from it. Every action transitions the environment to a new state and provides feedback in the form of rewards, which guide the agent’s learning process.

Value function: $V_{\pi }(s)$, describes *how good* it is to be in a specific state *s* under a certain policy *π*. For MDP:

2 $$\begin{aligned} V_{\pi }(s) = \mathbb {E}[G_t | S_t = s] \end{aligned}$$

To achieve the optimal value function, the WinRegRL agent will calculate the expected reward return when starting from *s* and following *π*, but account for the cumulative discounted reward.

3 $$\begin{aligned} V_*(s) = \max \limits _{\pi } V_\pi (s) \end{aligned}$$

WinRegRL agent at each step *t* receives a representation of the environment’s *state*, $S_t \in S$, and it selects an action $A_t \in A(s)$. Then, as a consequence of its action, the agent receives a *reward*, $R_{t + 1} \in R \in \mathbb {R}$.

Policy: WinRegRL *policy* is a mapping from a state to an action

4 $$\begin{aligned} \pi _t(a|s) \end{aligned}$$

That is the probability of selecting an action $A_t = a$ if $S_t = s$.

Reward: The total *reward* is expressed as:

5 $$\begin{aligned} G_t = \sum _{k = 0}^H \gamma ^kr_{t + k + 1} \end{aligned}$$

where *γ* is the *discount factor* and *H* is the *horizon*, which can be infinite.

### Employed RL technique

RL enables an autonomous system to learn from its experiences using rewards and punishments derived from its actions. By exploring its environment through trial and error, the RL agent refines its decision-making process^19^. As training RL agents requires a large number of samples from an environment to reach human-level performance^20^, we have opted for WinRegRL for an efficient approach by adopting model-based RL instead of training an agent’s policy network using actual environment samples. We only use dataset samples to train a separate model supervised by a certified WR Forensics expert, having acquired GCFE or GCFA. This phase enabled us to predict the environment’s behaviour, and then use this transition dynamics model to generate samples for learning the agent’s policy^21^.

**Fig. 3:**
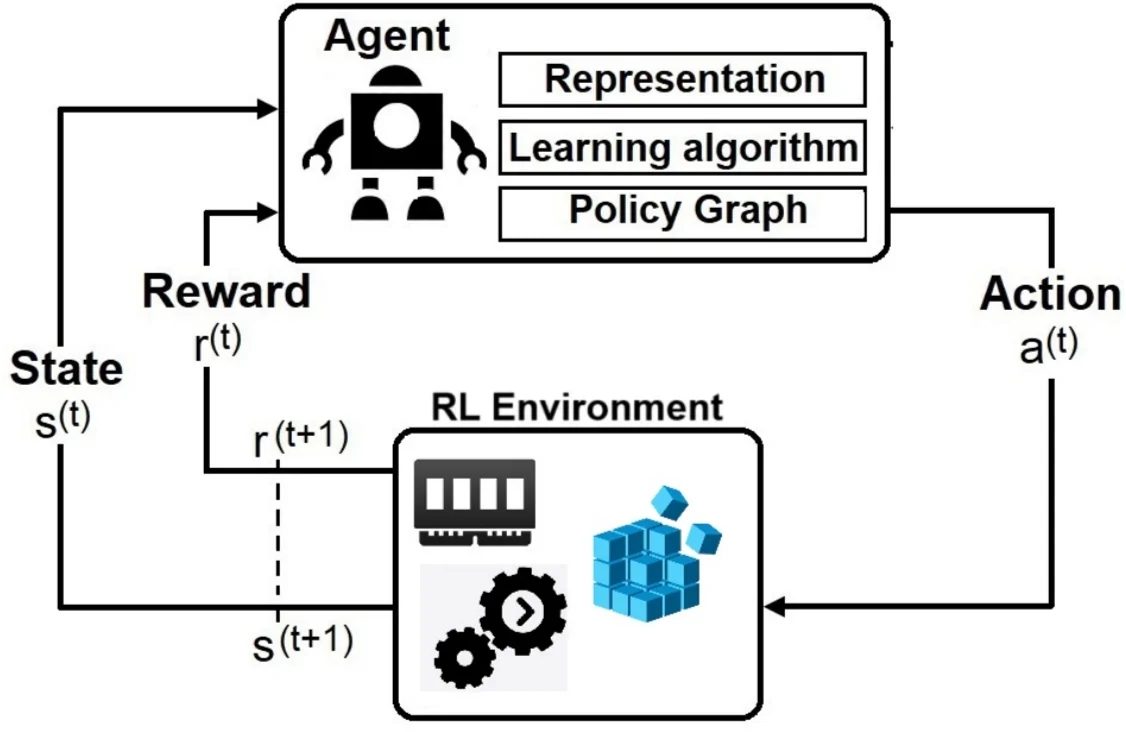
The RL conceptual framework in the context of WR and volatile data forensic analysis.

In this system, an RL agent interacts with its surround in a sequence of steps in time (*t*): (1) the agent receives a representation of the environment (state); (2) selects and executes an action; 3 receives the reinforcement signal; (4) updates the learning matrix; (5) observe the new state of the environment (Fig. 3).

The RL Agent at each step *t* receives a representation of the environment’s *state*, $S_t \in S$, and it selects an action $A_t \in A(s)$. Then, as a consequence of its action, the agent receives a *reward*, $R_{t + 1} \in R \in \mathbb {R}$. RL is about learning a policy (*Π*) that maximises the reinforcement values. A policy defines the agent’s behaviour, mapping states into actions. The *ε*-greedy method is an example of the action selection policy adopted in RL. We are seeking an exact and reproducible solution to our MDP problems and initially considered the following methods. WinRegRL is designed to act as a planning-and-refinement framework, model-based value/policy iteration is used when the specified transition model is sufficiently supported by empirical counts and expert priors, whereas online Q-learning is only invoked for out-of-support states or when unseen transition patterns are observed during evaluation. This makes the learning regime explicit and clarifies the operational conditions under which Q-learning is invoked.

#### Value iteration and action-value (Q) function

We denoted the expected reward for state and action pairs in Eq. (6).

6 $$\begin{aligned} q_{\pi }(s, a) = \mathbb {E}_{\pi }\begin{bmatrix} G_t | S_t = s, A_t = a \end{bmatrix} \end{aligned}$$

#### Optimal decision policy

WinRegRL optimal value-action decision policy is formulated by the following function:

7 $$\begin{aligned} q_*(s,a) = \max \limits _{\pi } q^\pi (s,a) \end{aligned}$$

We can then redefine $V^*$, Eq. (3), using $q^*(s,a)$, Eq. (7):

8 $$\begin{aligned} V_*(s) = \max \limits _{a \in A(s)} q_{\pi *}(s,a) \end{aligned}$$

This equation conveys that the value of a state under the optimal policy **must be equal** to the expected return from the best action taken from that state. Rather than waiting for V(s) to converge, we can perform policy improvement and a truncated policy evaluation step in a single operation. Algorithm 1 details the WinRegRL value iteration.

**Algorithm 1:**
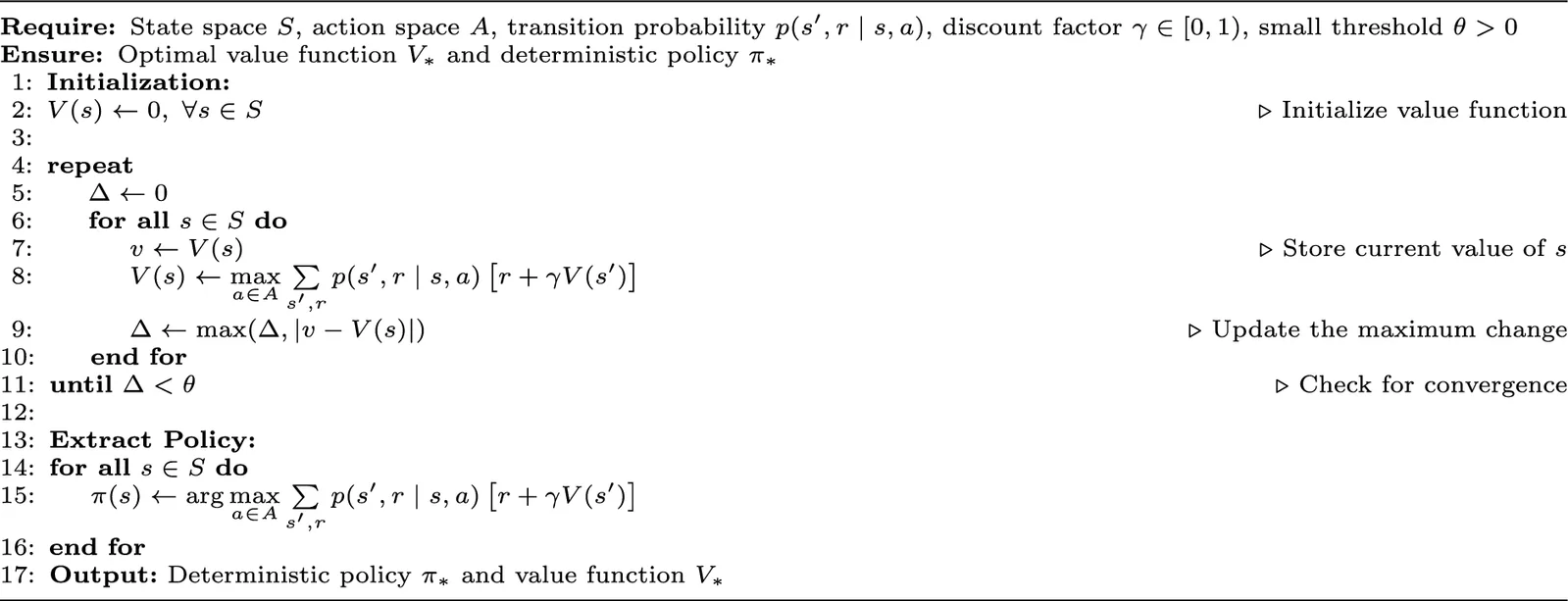
WinRegRL value iteration

Monte Carlo (MC) is a *model-free* method, meaning it does not require complete knowledge of the environment. It relies on **averaging sample returns** for each state-action pair^22^.

### Core algorithmic components

WinRegRL-Core: This module is the RL engine responsible for solving the formulated MDP efficiently. It alternates between *value iteration* and *Q-learning*, combining the convergence properties of model-based planning with the adaptability of model-free learning. Value iteration is applied in the early stages over the expert-specified state transition model, allowing for the rapid computation of an initial optimal policy. Q-learning is then employed to refine the policy when the model is incomplete or when the environment in which the investigation is conducted evolves dynamically. This hybrid strategy accelerates convergence and maintains optimal performance across varying forensic scenarios. WinRegRL-Core is a two-stage solver, Stage 1 performs model-based planning using the expert-specified transition matrix $\hat{P}$ and reward model *R* to compute an initial policy $\pi _0$. Stage 2 performs local Q-learning refinement only for low-support or sparsely observed state–action regions. The methodological interpretation is therefore kept strictly within the scope of tabular RL and dynamic programming.

**Fig. 4:**
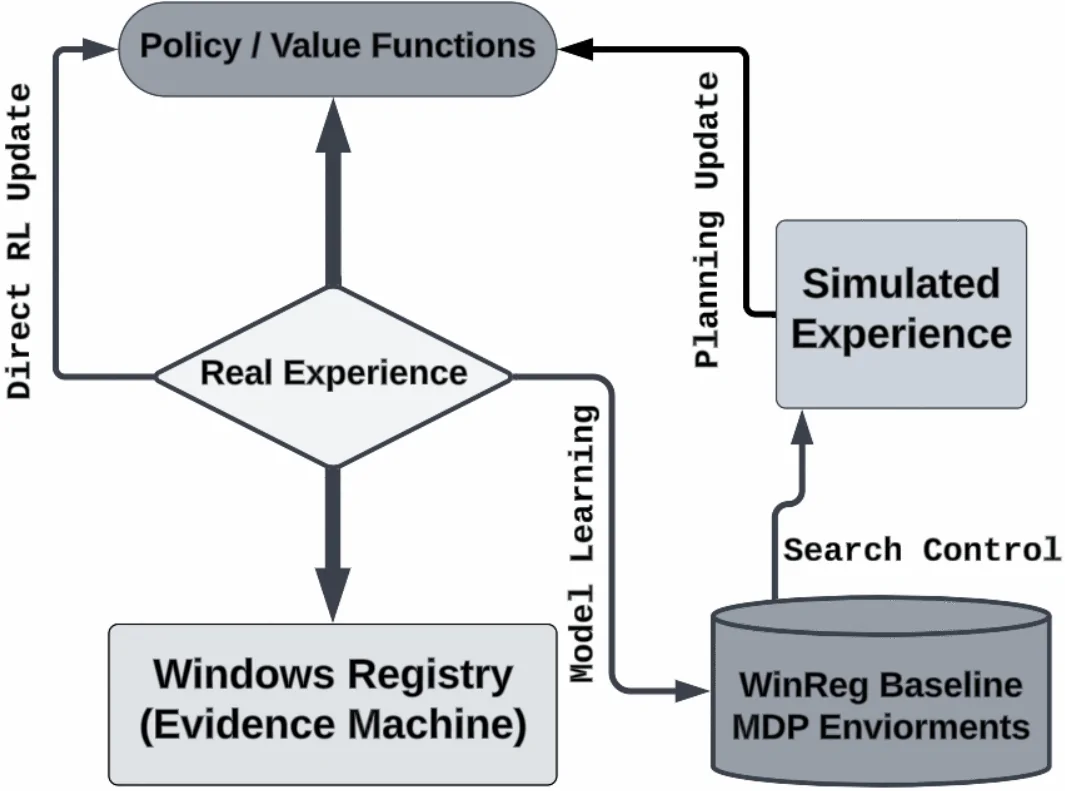
WinRegRL learning approach.

PGGenExpert-Core: This expert-in-the-loop module captures and generalises decision-making patterns from experienced digital forensic practitioners. It constructs *policy graphs* from sequences of expert actions taken during investigations, then abstracts and generalises these policies for storage in a knowledge base. These reusable strategies enable WinRegRL to leverage accumulated human expertise, allowing faster adaptation to similar or partially related cases in the future. The integration of PGGenExpert-Core ensures that the system benefits from both automated RL optimisation and the nuanced judgment of human experts. Expert trajectories were first normalised into a common action taxonomy and then converted into state—action visitation graphs. These graphs are used in two places: (i) as priors for transition specification and reward shaping, and (ii) as explainability artefacts that allow the resulting policy to be inspected against certified examiner reasoning. Figure 4 illustrates the sequential learning approach used in WinRegRL, showing the step-by-step interaction between the agent, environment, and policy update process.

#### Bellman equation

An important recursive property emerges for both Value (2) and Q (6) functions if we expand them^23^.

#### Value function

9 $$\begin{aligned} \begin{array}{l l} V_{\pi }(s)&= \sum \limits _{a} \pi (a | s) \sum \limits _{s'}\sum \limits _{r} p(s', r | s, a)\begin{bmatrix} r + \gamma V_{\pi }(s') \end{bmatrix} \end{array} \end{aligned}$$

Similarly, we can do the same for the Q function:

10 $$\begin{aligned} \begin{array}{l l} q_{\pi }(s,a)&=\sum \limits _{s',r} p(s', r| s, a)\begin{bmatrix}r +\gamma V_{\pi }(s') \end{bmatrix}\\ \end{array} \end{aligned}$$

#### Policy iteration

The optimal policy generated by WinRegRL is summarised and presented in Algorithm 2.

**Algorithm 2:**
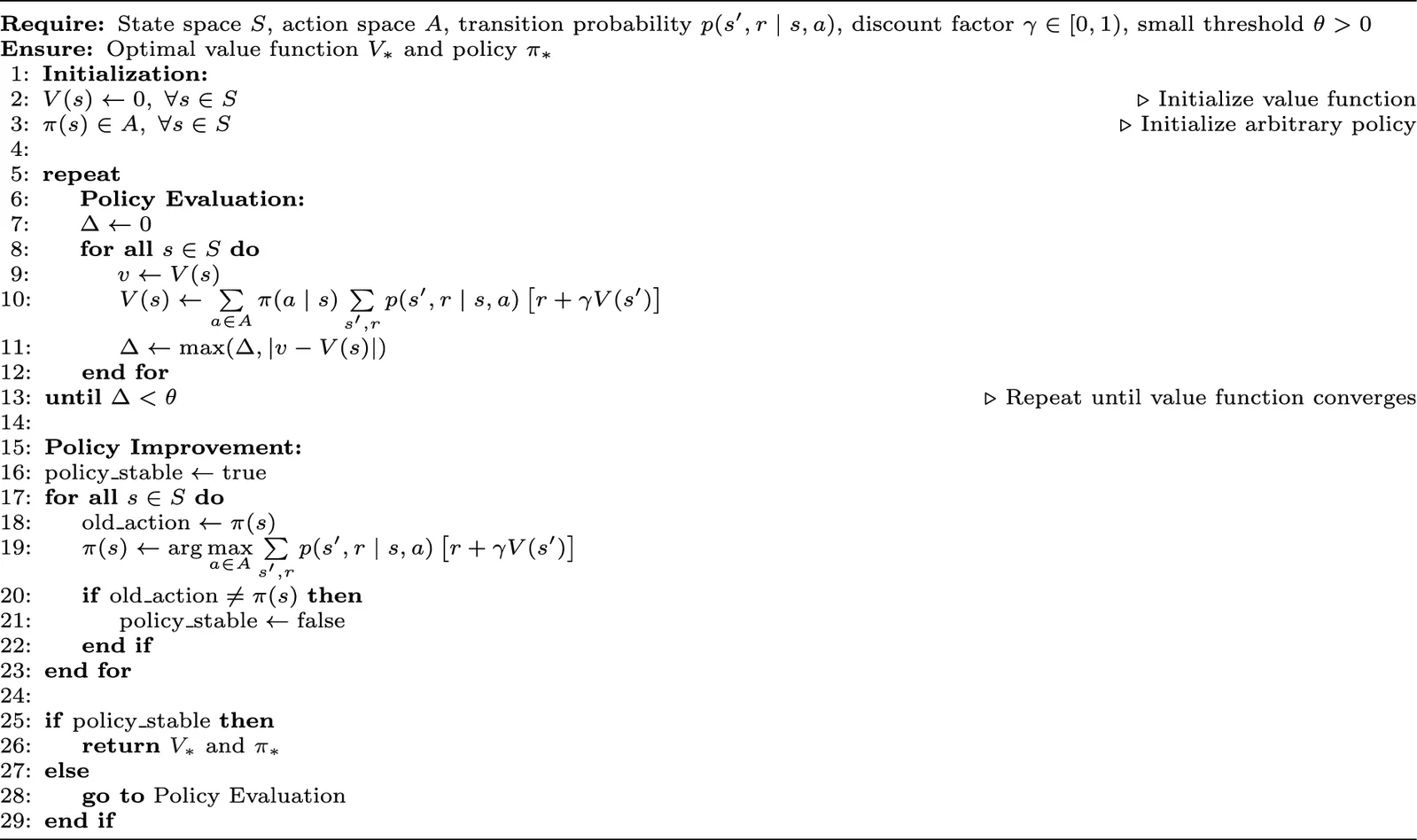
WinRegRL policy iteration

### WinRegRL framework design and implementation

**Fig. 5:**
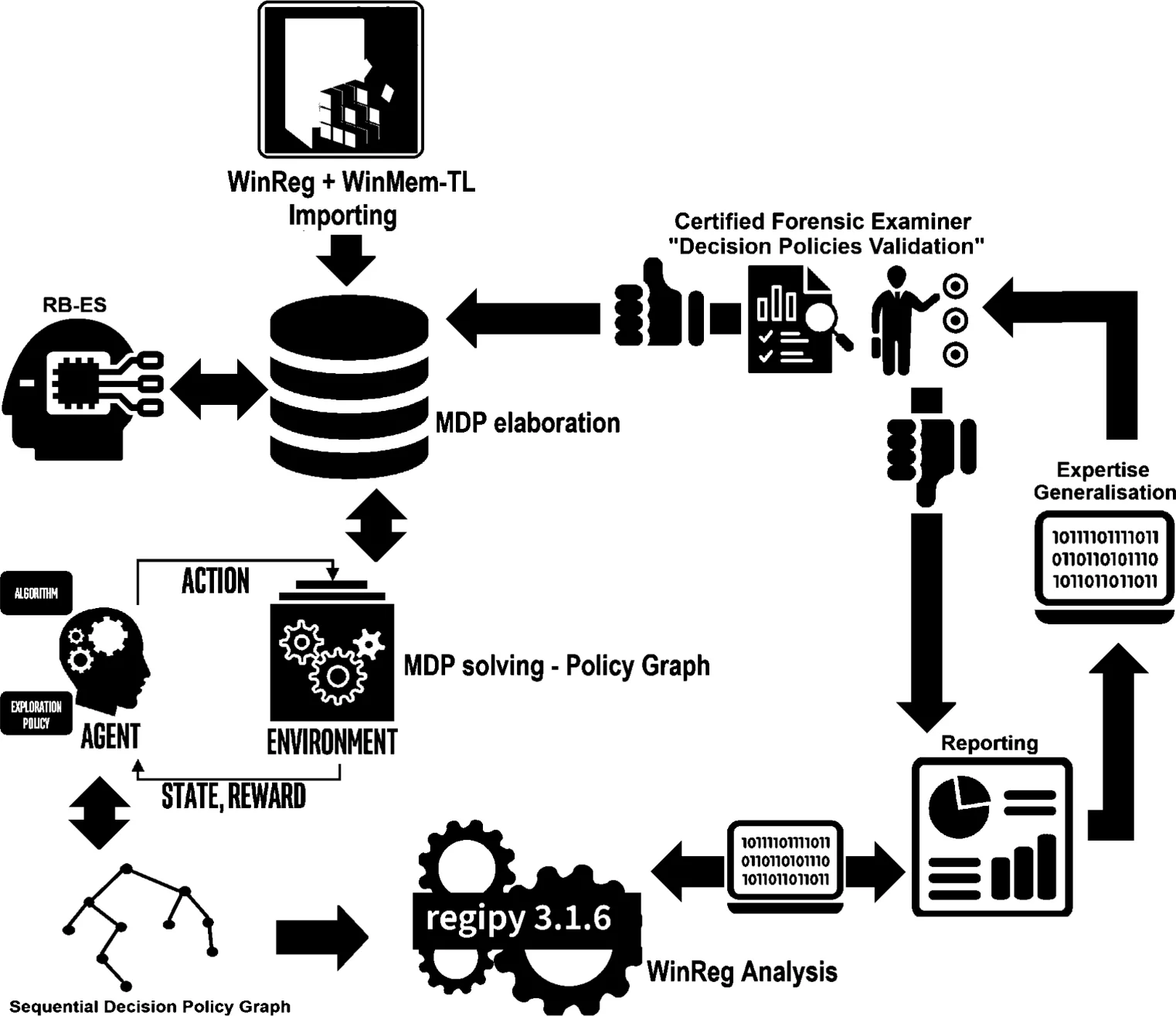
WinRegRL framework detailed functioning diagram.

Figure 5 provides a detailed diagram illustrating the functioning of WinRegRL, covering WR hives and volatile data parsing, MDP environment elaboration, RL and RB-AI operation, as well as human expert (GCFE) validation. The diagram only considers WR analysis and volatile memory (WinMem-TL) data for elaborating MDP envisionments. This framework highlights an iterative decision-making process that combines expert knowledge with RL and automated forensic tools to enhance the analysis and detection of fraudulent or anomalous activities in a Windows environment. The modular breakdown of the WinRegRL framework is presented here:

WinReg + WinMem-TL importing: The process begins by importing WR data and memory timelines (WinMem-TL).

MDP elaboration: The imported data is transformed into an MDP environment following the structure defined in Equation 1.

MDP solver: WinRegRL uses a finite-state discrete-action solver implemented in MATLAB R2024a using the MDPtoolbox dynamic-programming routines for value iteration and policy extraction. The operative configuration used for the reported experiments is fixed and auditable: discount factor *γ =0.99*, value-iteration tolerance $\epsilon =10^{-5}$, local-refinement support threshold *τ =0.10*, maximum horizon *H=200*, and at most 200 Bellman sweeps before policy extraction. The reported results are obtained using a finite-state discrete-action solver without auxiliary neural optimisation modules. Instead, WinRegRL first solves the expert-specified model $\left( S,A,\hat{P}, R,\gamma \right)$ by dynamic programming and only then invokes local tabular Q-learning in low-support regions. The algorithm functioning is detailed in “ WinRegRL-Core”.

### Expertise capturing and generalisation

An RB-AI with an online integrator that interacts with the RL MDP-solver, likely applying expert knowledge or predefined rules to assist in elaborating decision policies based on the imported data^24^. If the generated policies are inadequate, they undergo further *expertise generalisation*, where more complex or nuanced decision-making logic is applied to better handle fraud detection or anomaly identification.

#### Reporting and analysis

The validated results are compiled into a report for decision-making or further investigation. The process feeds back into WinReg analysis using Regipy 3.1.6, a tool for forensic analysis of WR hives, ensuring continual refinement and improvement of the decision-making model.

### Human expert validation module

The generated decision policies are validated by a certified forensic examiner holding at least GIAC GCFE or GCFA to ensure that the automatically generated decision policies are correct and appropriate. If the policies are valid, they receive a “thumbs up”; if not, further generalisation or adjustments are needed (denoted by the “thumbs down” icon).

### WinRegRL-Core

WinRegRL-Core, as illustrated in Algorithm 3, is the main decision engine for the finite MDP constructed from the forensic investigation space. The algorithm is formulated as a *two-stage hybrid solver*. In Stage 1, the expert-defined transition model $\hat{P}$ and reward model *R* are solved by value iteration until the Bellman residual satisfies $\Vert V_{k+1}-V_k\Vert _{\infty }<10^{-5}$. The resulting greedy policy is then extracted as the nominal forensic policy $\pi ^\star$. In Stage 2, local tabular Q-learning is activated *only when needed*, namely when the selected state–action pair has empirical support below the threshold *τ =0.10*, when an unseen branch is encountered, or when the observed transition deviates from the expert prior beyond the tolerance used during validation. Under these conditions, the agent performs bounded $\epsilon _g$-greedy exploration around the planned action and updates the tabular *Q*-values for that local region only. Q-learning is not a second full training regime, but a bounded refinement mechanism for sparse or uncertain regions of the state space. Convergence is therefore defined purely through dynamic-programming optimality and, where local refinement is invoked, through stabilisation of the local *Q*-value ranking rather than through an arbitrary target-policy threshold such as 0.1.

**Algorithm 3:**
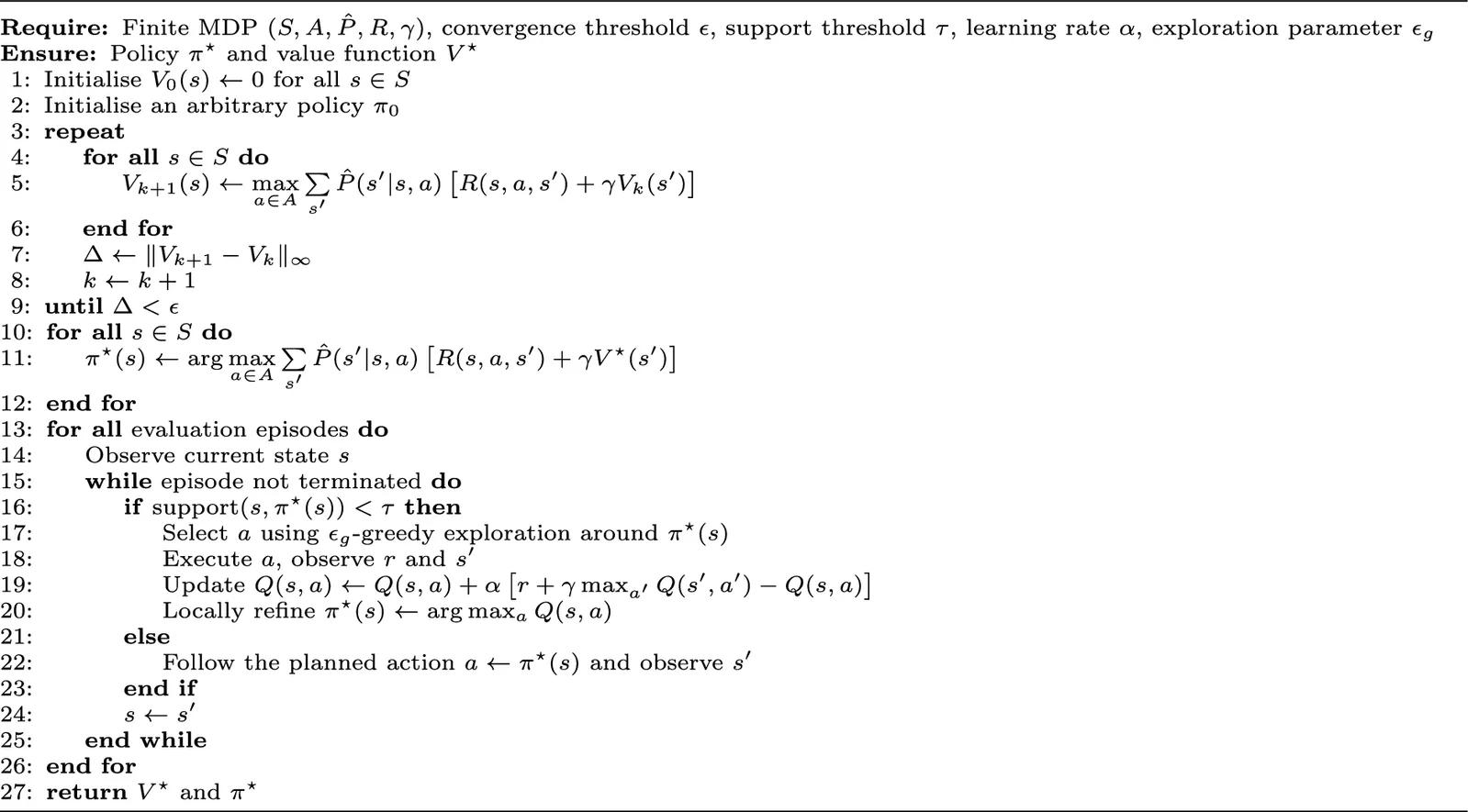
WinRegRL-Core hybrid planner and local refiner

Algorithm 3 therefore highlights that the Q-learning is only needed for low-support or out-of-distribution branches, identified operationally by $\mathrm {support~}(s, a)<\tau$. Moreover, it clearly states that all reported results use a discrete MDP solver with optional local tabular refinement. Finally, the algorithm’s convergence threshold is defined by mathematically standard stopping criteria based on the Bellman residual and policy stability.

For non-stationary problems, the Monte Carlo update rule for, e.g., *V* is:

11 $$\begin{aligned} V(S_t) \leftarrow V(S_t) + \alpha \begin{bmatrix} G_t - V(S_t) \end{bmatrix} \end{aligned}$$

Where *α* is the learning rate, how much we want to forget about past experiences.

### PGGenExpert-Core

PGGenExpert (Algorithm 4) is a deterministic policy-graph correction and generalisation routine that converts a case-specific WinRegRL policy graph into a normalised, expert-consistent policy graph for reuse on future cases. We emphasise that PGGenExpert is a post-processing and knowledge-consistency step that operates on an already-solved policy graph; it is not a learning algorithm in itself and does not, on its own, claim novel inferential power. Its role is to enforce three properties that make the policy graph admissible as a transition prior for the MDP solver and auditable by a certified examiner. It takes as inputs the original WR feature matrix, a complementary volatile-data matrix, the RL-derived state-to-action policy graph, and an expert-corrected reference policy graph. The routine then (i) bounds ordinal action values to the admissible range observed in the data, correcting out-of-range entries; (ii) repairs invalid binary actions by substituting expert-provided corrections where the volatile-data and Registry-derived values disagree; and (iii) collapses each state’s action distribution to its single highest-utility action, yielding a sparse, deterministic policy graph. The resulting graph is returned and used to update the MDP transition priors, ensuring that the reused policy remains consistent with expert knowledge and domain constraints while being directly inspectable by a human examiner.

**Algorithm 4:**
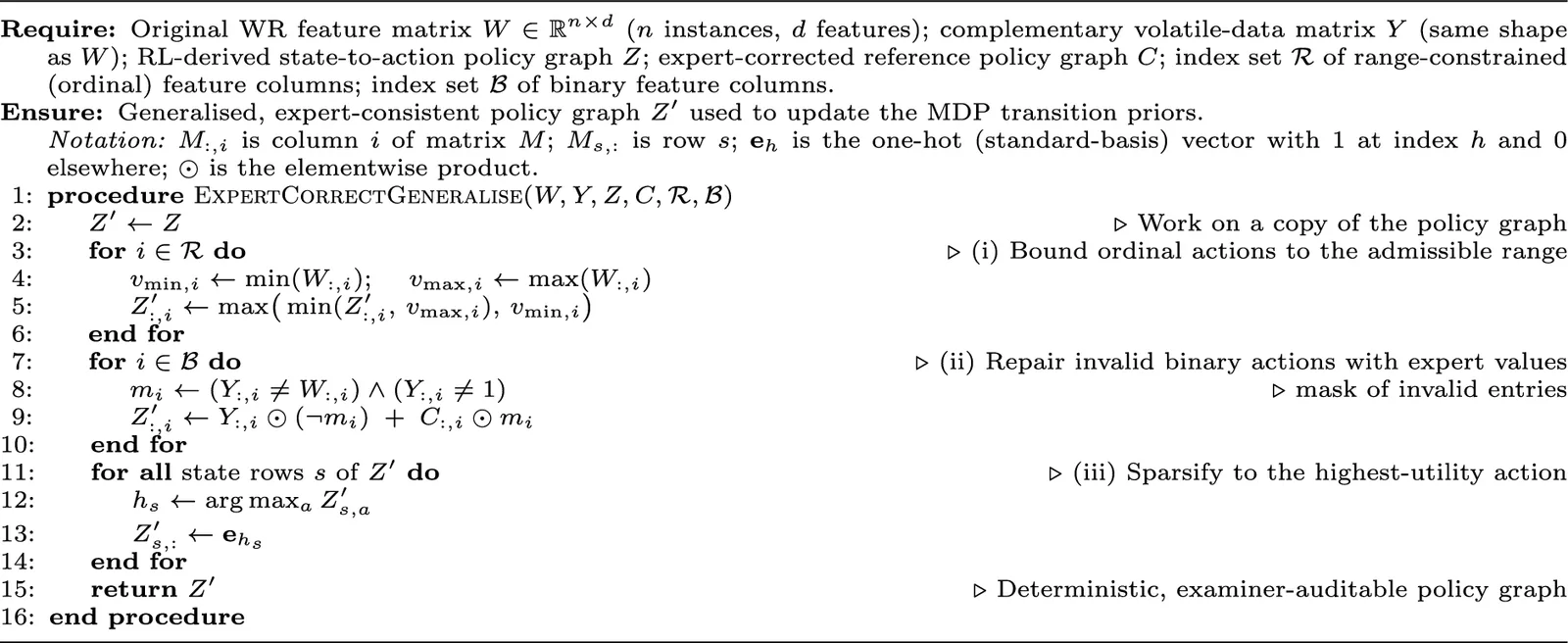
PGGenExpert: policy-graph expert correction and generalisation

## Testing, results and discussion

### Evaluation protocol, ground truth, and reproducibility

For each dataset *d*, we define a ground-truth artefact set $G_d$ containing adjudicated relevant artefacts associated with the investigated scenario. Ground truth was established by combining: (i) published challenge solutions or organiser write-ups where available, (ii) manual verification by a GCFE/GCFA-guided examiner, (iii) cross-source corroboration using Registry, volatile-memory, and timeline evidence, and (iv) duplicate removal and normalisation at the artefact-instance level so that semantically identical findings reported through different tools are counted only once. Returned artefacts from a method *m* are denoted by $A_{m,d}$ and scored at the artefact-instance level rather than only at the category level. To make the evaluation statistically interpretable, each WinRegRL benchmark was repeated over 10 independent runs per dataset using different random seeds for the local refinement stage, yielding 40 WinRegRL runs in total across the four datasets. Hyperparameters, reward weights, discount factor, and support thresholds were frozen before final benchmarking and were not re-tuned per dataset. Because the main planning stage is deterministic once $\hat{P}$ and *R* are fixed, the observed variance arises primarily from the bounded $\epsilon _g$-greedy local refinement stage and from the timing variability of external parsing utilities.

### Controlled comparative design

To avoid an unfair comparison between a fully automated framework and interactive forensic utilities, we separate the comparative evaluation into two settings. In the first setting, WinRegRL is compared with FTK Registry Viewer and KAPE under analyst-assisted scripted workflows using the same forensic inputs, the same hardware environment, and the same stopping criteria. In the second setting, WinRegRL output is compared with examiner-led investigations conducted by certified analysts under a controlled time budget and with access to the same evidential inputs. The human study protocol is now made explicit: three certified examiners (two GCFE and one GCFA, each with more than five years of Windows forensic casework) completed the same four case tasks in counter-balanced order, with a 90-minute limit per case, no collaboration between participants, and access to the same acquired Registry, memory, and timeline inputs made available to the automated baselines. Their outputs were scored against the same adjudicated benchmark set $G_d$.

### Formal metric definition

We define the accuracy and coverage measures formally. At the artefact-instance level, a true positive (*TP*) is an artefact returned by a method that is present in the adjudicated benchmark set $G_d$ for the investigated case; a false positive (*FP*) is a returned artefact not supported by the benchmark after adjudication; and a false negative (*FN*) is a benchmark artefact missed by the method. Because the experiment scores retrieved evidence items rather than an exhaustive universe of irrelevant artefacts, true negatives are not informative and are therefore not used as the principal headline metric. Artefact-level precision is computed as

$$\textrm{Precision}_{m,d} = \frac{|A_{m,d}\cap G_d|}{|A_{m,d}|} = \frac{TP}{TP+FP},$$

artefact-level recall (coverage) is computed as

$$\textrm{Recall}_{m,d} = \frac{|A_{m,d}\cap G_d|}{|G_d|} = \frac{TP}{TP+FN},$$

and the harmonic mean is

$$F1_{m,d} = \frac{2\cdot \textrm{Precision}_{m,d}\cdot \textrm{Recall}_{m,d}}{\textrm{Precision}_{m,d}+\textrm{Recall}_{m,d}}.$$

For completeness, when a closed candidate list of artefacts is available for a case, the corresponding case-bounded accuracy may be computed as *(TP+TN)/(TP+TN+FP+FN)*; The precision, recall, and F1-score choice is judged as more appropriate for retrieval-oriented forensic tasks. In addition, time-to-completion, time-to-first-relevant-artefact, and category-level recall were tracked during benchmarking. This formalisation ensures that evaluation is not self-referential and that high coverage refers only to recall against the adjudicated benchmark for the evaluated case, rather than to a universal claim of completeness in arbitrary real-world incidents.

### Statistical analysis

For WinRegRL, FTK, and KAPE, completion times were summarised as mean±standard deviation across repeated runs, and 95% confidence intervals for precision, recall, and F1 were computed using non-parametric bootstrap resampling (2000 resamples) over adjudicated artefact instances. For the human-expert study, median and interquartile range were additionally retained because examiner performance is inherently heterogeneous. Pairwise differences in completion time and recall were assessed using a paired Wilcoxon signed-rank test across case-level runs, and effect sizes were summarised using Cliff’s *δ*. This statistical layer was added specifically to avoid reliance on single-point claims and to make the observed benchmarking margins quantitatively interpretable.

### Human-expert protocol

Participating examiners were given the same input artefacts, the same case scope, and the same completion criteria. Their task was to identify and justify scenario-relevant Registry and correlated timeline artefacts. The study records the number of relevant artefacts found, unsupported artefacts reported, time to completion, and evidential justification quality.

### Testing datasets

To test WinRegRL, we opted for different DFIR datasets. Table 8 summarises the WinRegRL testing datasets, which were a mixed selection of four (04) datasets that cover multiple Windows architectures and versions. In general, three recent datasets are widely adopted in DFIR research, respectively Magnet-CTF (2022), IGU-CTF (2024) and MemLabs-CTF (2019); each dataset is individually referenced in Table 8. We note for transparency that the fourth dataset, MalVol-25, was co-developed by some of the present authors; to avoid any self-referential evaluation bias, the ground truth for MalVol-25 was adjudicated using the same external protocol applied to the three independent datasets, and the dataset is used only as one of four heterogeneous test sets. As reported in Table 10, WinRegRL’s margin over the FTK and KAPE baselines is in fact *smallest* on MalVol-25, so the headline comparative findings are driven by the three independently authored datasets rather than by the in-house dataset.

**Table 8:** WinRegRL testing forensics datasets.

Datasets	Description
Magnet-CTF 2022^25^	Dataset developed by Magnet Forensics, including several forensic images as a public service part of Magnet CTF-2022. Datasets made of Registry and Memory of different Windows Machines (10, 8, 7 and XP)
IGU-CTF 2024^26^	Dataset was taken from IGUCTF-24 which include CVE-2023-38831 Lockbit 3.0 Ransomware. The dataset is made of the Registry and Memory of infected Windows machines, capturing Powershell2exe and Persistence tactics
MemLabs-CTF 2019^27^	Dataset developed by MemLabs with CTF Registry and Memory Forensics. All the labs are on Windows 7. Datasets made of Registry and Memory of Six (06) different Windows 7 Machine.
MalVol 2025^28^	IEEE Diverse, Labelled and Detailed Malware Volatile Memory Dataset for Detection and Response Testing and Validation, Files are **.mem** and **.raw** and include full system Registry and RAM dump

### RL parameters and variables

In this subsection, we report on parameters and variables that are actually active in the final solver. In particular, we focus on discount factor, planning horizon, Bellman-residual tolerance, local-support threshold, and bounded Q-learning exploration settings. Reward magnitudes are no longer described generically as “empirical” but are tied directly to the forensic utility schedule in Table 7, where high values correspond to corroborated case-critical evidence and negative values correspond to redundant or invalid investigative actions. Parameters not used by the finite-state hybrid planner are explicitly marked as removed from the final reported configuration so that the solver description remains aligned with the actual implementation. Table 9 summarises the exact configuration used in the experiments.

**Table 9:** Final WinRegRL solver configuration used for the reported experiments. Parameters formerly shown only as broad ranges are now replaced by the exact instantiated values used during benchmarking.

Parameter	Final value used	Description
*γ*	0.99	Discount factor used in all reported experiments
*H*	200	Maximum number of decision epochs allowed per episode
*ε*	$10^{-5}$	Bellman-residual stopping tolerance for value iteration
*τ*	0.10	Minimum empirical support threshold below which local Q-learning refinement is triggered
*α*	0.05	Learning rate for the local tabular Q-learning refinement stage
$\epsilon _g$ (initial)	0.10	Initial exploration probability for low-support local refinement
$\epsilon _g$ (final)	0.02	Final exploration probability after decay within the bounded local refinement stage
$\mathcal {T}$	$10^{-3}$	Transition-deviation tolerance used to flag unstable branches for local refinement
Max Bellman sweeps	200	Upper limit on dynamic-programming iterations before forced policy extraction
Bootstrap resamples	2,000	Number of resamples used for confidence-interval computation
Random seeds	10	Independent seeds used for repeated WinRegRL runs per dataset
Reward scale	*[-10,+100]*	Operational reward interval; exact category-specific assignments are given in the reward schedule table

### WinRegRL results

**Fig. 6:**
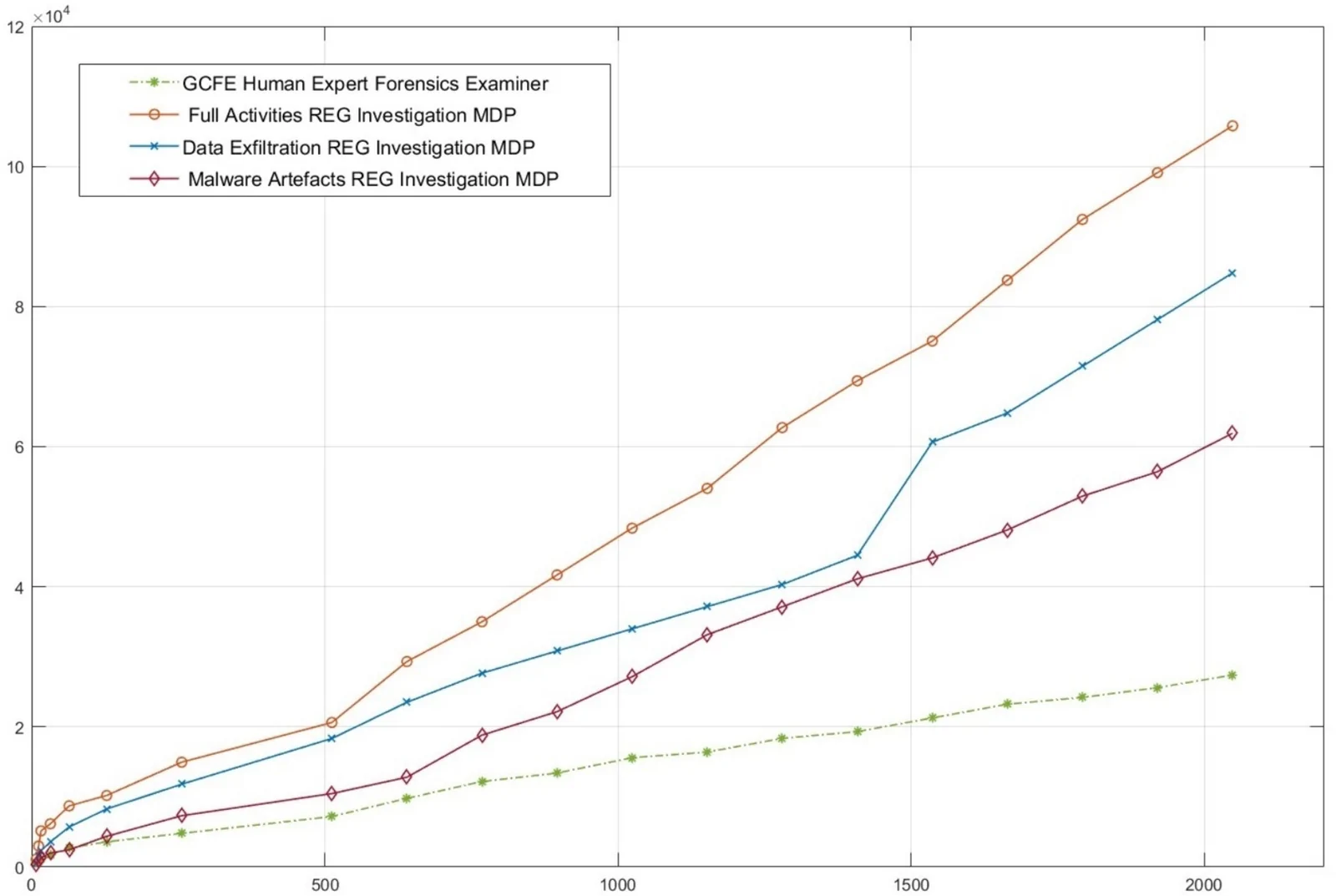
Results solving MDP problem in different environments.

Figure 6 illustrates the evaluation of the WinRegRL framework’s performance in different scenarios of MDPs, including the full activities REG investigation MDP, the Data Exfiltration REG Investigation MDP, and the Malware Artefacts REG Investigation MDP. It is contrasted with the baseline time taken by a GCFE Human Expert Forensics Examiner (green dashed), used only as a reference point. The horizontal axis represents the WR size in MB, while the vertical axis represents the time in seconds it took to run the respective MDP tasks. The results show that Full Activities REG Investigation MDP always takes the longest time, followed by Data Exfiltration REG Investigation MDP, then Malware Artefacts REG Investigation MDP—showing how different the task complexities are. Notably, the GCFE Human Expert shows a linear and considerably lower time requirement in smaller datasets but points out scalability limitations when dealing with larger registries.

**Fig. 7:**
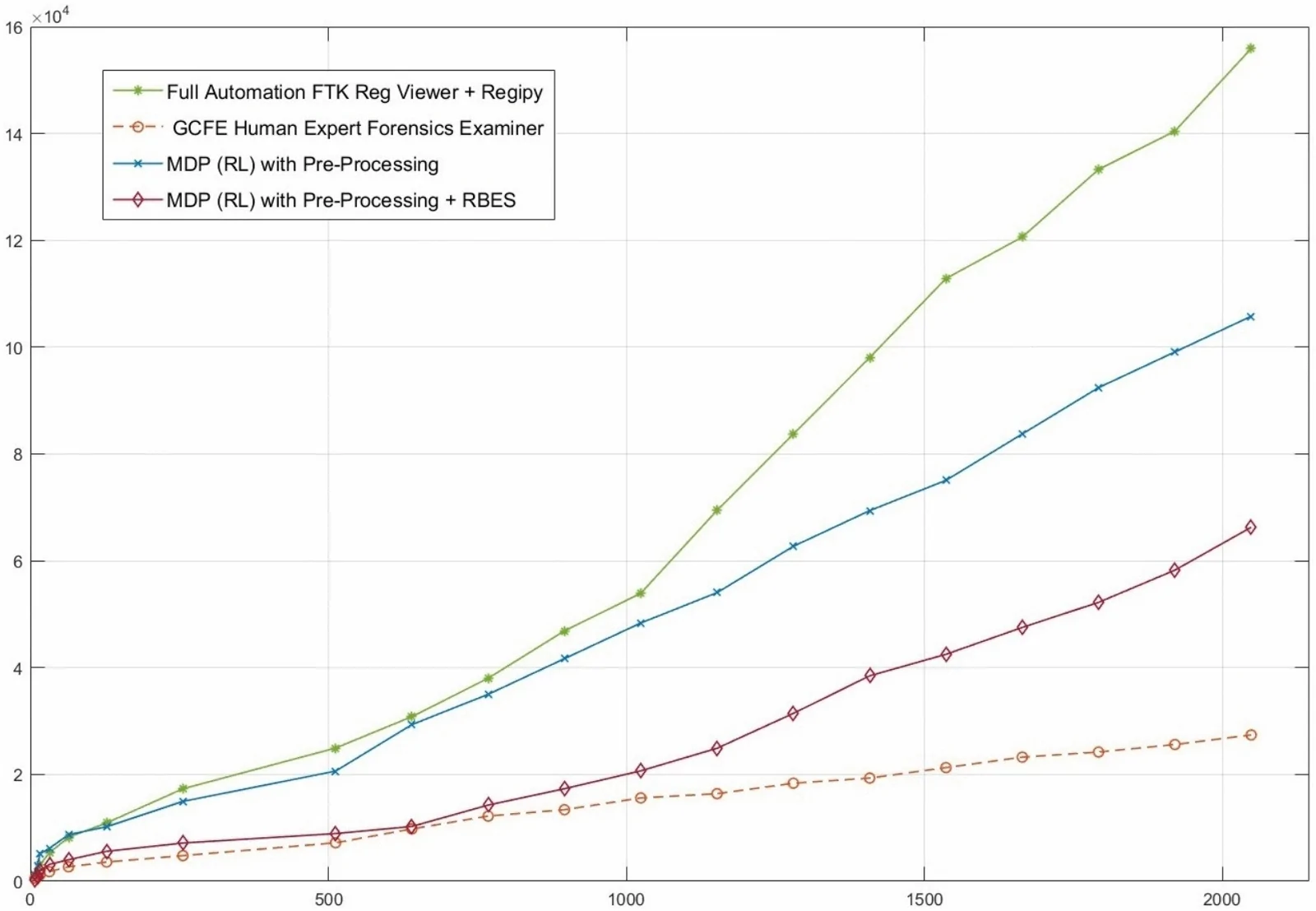
WinRegRL automation results.

On the other hand, Fig. 7 presents a comparative analysis of the WinRegRL framework’s performance with respect to different WR sizes (x-axis) and the time required to complete forensic analyses (y-axis, in seconds). Four approaches are compared: Full Automation FTK Reg Viewer with Regipy, GCFE Human Expert Forensics Examiner, MDP (RL) with pre-processing, and MDP (RL) with pre-processing and RB-AI. The full WinRegRL pipeline remains the fastest approach in the tested scenarios, and the gain becomes more pronounced as Registry size increases; however, this is reported as a controlled case-study observation rather than as an unconditional statement that automation always surpasses expert-led practice. The pre-processing-only RL variant shows moderate scalability, while the addition of RB-AI materially improves prioritisation and therefore reduces completion time. The examiner’s baseline remains slower on the largest cases, but its value lies in evidential interpretation rather than raw speed alone.

**Fig. 8:**
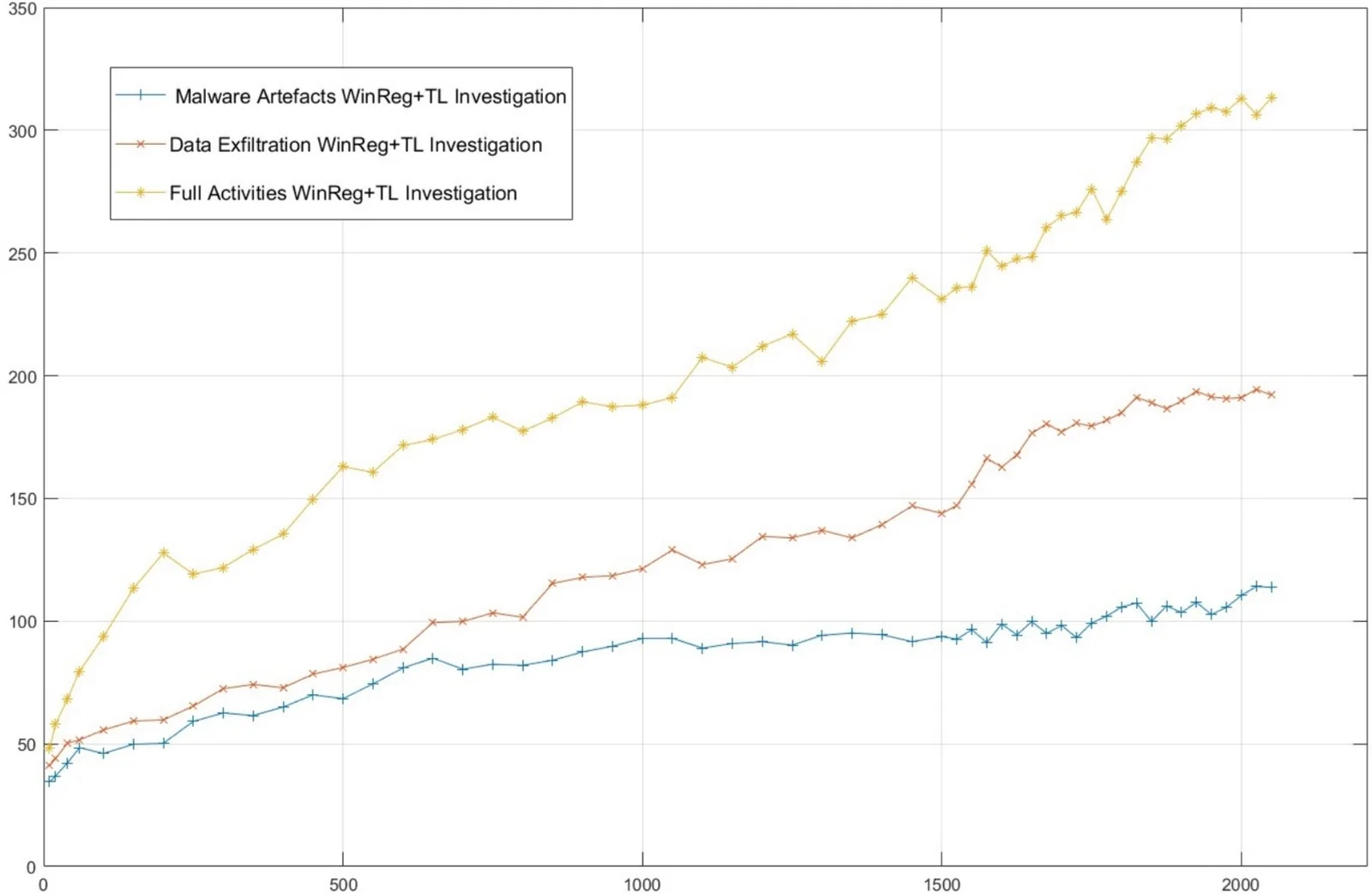
Overall number of relevant artefacts processed in a variable-size Registry hives with a maximum value of 2048 Mb.

**Fig. 9:**
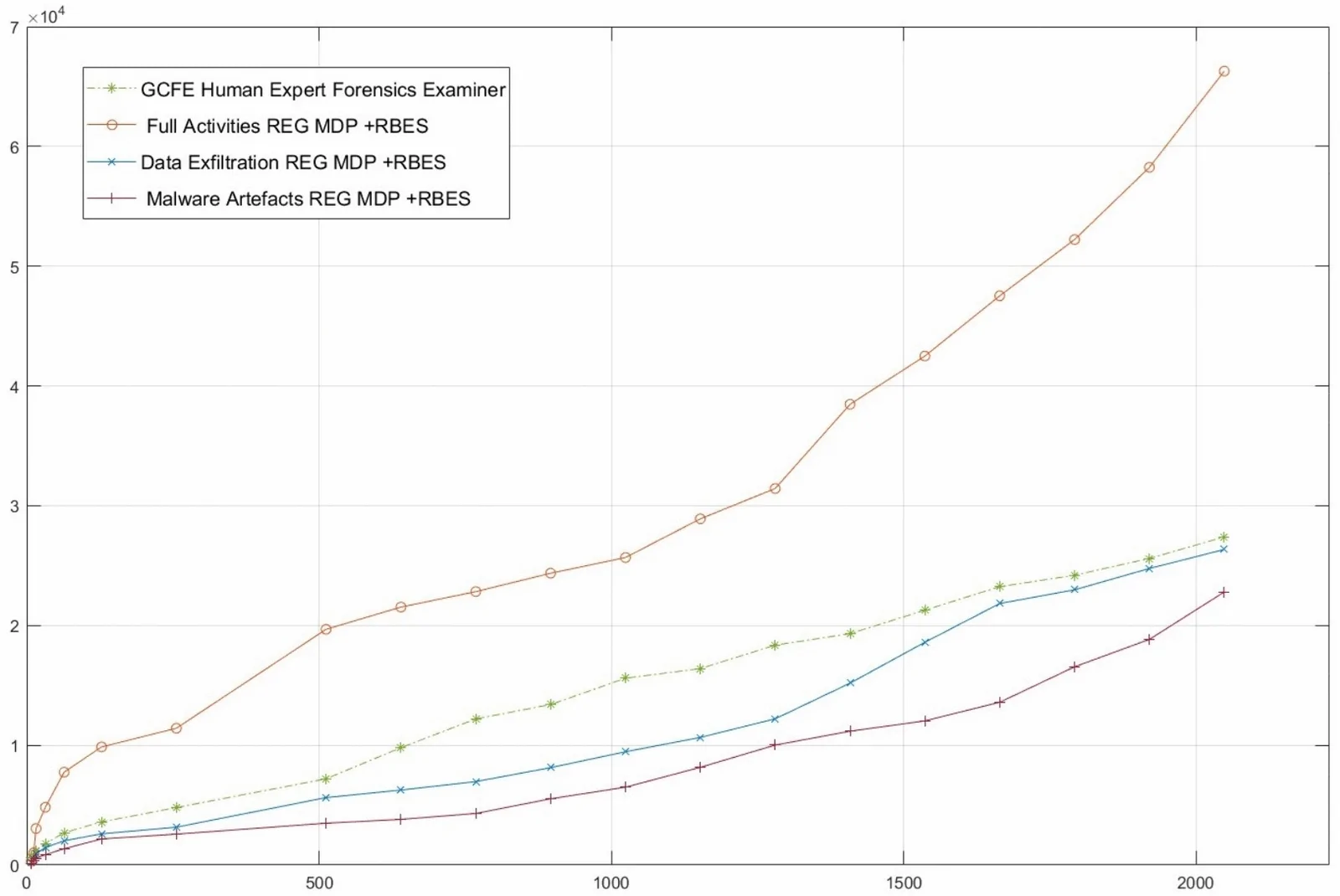
Execution times of WinRegRL and human experts.

Figure 8 illustrates the efficacy of the WinRegRL Framework across three investigation scenarios: Malware Artefacts, Full WR with Timeline (WinReg+TL) Investigation, Data Exfiltration WinReg+TL Investigation, and full activities WinReg+TL Investigation. The x-axis represents WR size, while the y-axis indicates the number of artefacts discovered in each case. Results show that the Full Activities WinReg+TL Investigation consistently uncovers the highest number of artefacts, increasing rapidly as registry size grows. The Data Exfiltration WinReg+TL Investigation follows, with a steady rate of artefact discovery. In contrast, the Malware Artefacts WinReg+TL Investigation yields the fewest artefacts, with slower growth and a plateau at larger registry sizes. These findings suggest that both the complexity and quantity of artefacts vary across investigation types, highlighting that full-activity investigations cover a broader scope than targeted malware or data theft analyses.

Figure 9 illustrates the performance of the WinRegRL framework tested under varying WR sizes (x-axis) against execution time in seconds (y-axis) across three scenarios: full activities, data exfiltration, and malware artefacts, in comparison to a human expert (GCFE). The results demonstrate that while the GCFE maintains relatively stable performance on smaller and moderately sized cases, WinRegRL becomes increasingly time-efficient as Registry size and evidential complexity grow. The full activities scenario exhibits the largest time-efficiency gain, followed by data exfiltration and malware artefacts. These figures are treated as evidence of scalability under the tested protocol rather than as proof of universally error-free operation or categorical replacement of human expertise.

### Formal artefact-level evaluation and confusion-matrix formulation

To eliminate ambiguity regarding the terms “accuracy” and “coverage”, the manuscript evaluates WinRegRL at the level of *candidate forensic artefact instances* rather than over the full Windows Registry. This distinction is essential because a raw Registry can contain millions of keys and values, the overwhelming majority of which are not evidentially relevant to a given incident. Scoring over the full Registry would therefore make true negatives trivially dominant and artificially inflate accuracy.

Accordingly, for each dataset *d*, we define a bounded candidate artefact universe $U_d$ containing only artefact instances drawn from the *forensically relevant Registry locations and associated corroborative traces* identified through SANS-oriented Windows Registry forensic guidance and the expert-derived investigation taxonomy used in WinRegRL. Each candidate artefact instance is represented as

$$u = (h, p, v, c, \tau , \kappa ),$$

where *h* denotes the source hive or evidence source, *p* the artefact path or key location, *v* the value name or artefact identifier, *c* the extracted content, *τ* the associated timestamp or temporal context, and *κ* the artefact category (e.g., execution, persistence, device usage, recent activity, or cross-source corroboration).

Let $G_d \subseteq U_d$ denote the adjudicated ground-truth set of relevant artefact instances for case *d*, established from challenge solutions where available, examiner validation, and manual adjudication against the case narrative. Let $A_{m,d} \subseteq U_d$ denote the set of artefact instances returned by method *m* on dataset *d*. We then define:

12 $$\begin{aligned} TP_{m,d} = |A_{m,d} \cap G_d|, \end{aligned}$$

13 $$\begin{aligned} FP_{m,d} = |A_{m,d} \setminus G_d|, \end{aligned}$$

14 $$\begin{aligned} FN_{m,d} = |G_d \setminus A_{m,d}|, \end{aligned}$$

15 $$\begin{aligned} TN_{m,d} = |U_d| - TP_{m,d} - FP_{m,d} - FN_{m,d}. \end{aligned}$$

Under this formulation, a true positive is a relevant artefact instance correctly identified by the method; a false positive is an artefact instance reported as relevant but not supported by the adjudicated benchmark; a false negative is a benchmark artefact missed by the method; and a true negative is a candidate artefact instance within the bounded SANS-scoped search space that is correctly not flagged as relevant. Importantly, true negatives are *not* computed over the entire Registry, but only over the bounded candidate artefact universe $U_d$, thereby preventing misleadingly inflated scores.

The principal retrieval-oriented metrics are defined as follows:

16 $$\begin{aligned} \textrm{Precision}_{m,d} = \frac{TP_{m,d}}{TP_{m,d}+FP_{m,d}}, \end{aligned}$$

17 $$\begin{aligned} \textrm{Recall}_{m,d} = \frac{TP_{m,d}}{TP_{m,d}+FN_{m,d}}, \end{aligned}$$

18 $$\begin{aligned} F1_{m,d} = \frac{2 \cdot \textrm{Precision}_{m,d} \cdot \textrm{Recall}_{m,d}}{\textrm{Precision}_{m,d}+\textrm{Recall}_{m,d}}, \end{aligned}$$

19 $$\begin{aligned} \textrm{Accuracy}_{m,d} = \frac{TP_{m,d}+TN_{m,d}}{TP_{m,d}+TN_{m,d}+FP_{m,d}+FN_{m,d}}. \end{aligned}$$

In this study, “artefact coverage” is defined operationally as recall against the adjudicated benchmark set $G_d$ and is therefore not self-referential. To further reduce the risk that large artefact families dominate the score, we additionally track category-level recall:

20 $$\begin{aligned} \textrm{CatRecall}_{m,d} = \frac{1}{K}\sum _{k=1}^{K} \frac{|A_{m,d} \cap G_{d}^{(k)}|}{|G_{d}^{(k)}|}, \end{aligned}$$

where $G_d^{(k)}$ is the ground-truth subset for artefact category *k* and *K* is the number of forensic categories considered in the case.

Because digital forensic triage is inherently time-sensitive, quality metrics were paired with temporal utility measures. Specifically, we report (i) time-to-first-relevant-artefact, (ii) total case completion time, and (iii) cumulative recall as a function of elapsed time. This allows the comparison with FTK, KAPE, and human examiners to be interpreted not only in terms of the final number of artefacts recovered, but also in terms of how rapidly useful evidential coverage is achieved.

### WinRegRL performances validation

Finally, the obtained results were validated by running the same experiments on different forensic datasets. WinRegRL precision, recall, F1-score, and completion-time benchmarking, compared with industry tools, are summarised in Table 10. The presentation reports mean performance for WinRegRL over repeated runs and retains the baseline FTK Registry Viewer and KAPE values as fixed scripted-workflow references. The overall trend remains clear: under the controlled protocol, WinRegRL recovered a larger proportion of adjudicated relevant artefacts than the two automated baselines while maintaining substantially higher precision.

**Table 10:** Artefact-level precision and recall benchmarking of WinRegRL against FTK Registry Viewer and KAPE across four forensic scenarios. Reported recall corresponds to coverage against the adjudicated benchmark set for each case.

Tool or framework	Artefact-level precision			Artefact-level recall (coverage)		
	**Our**	FTK RV	KAPE	**Our**	FTK RV	KAPE
Magnet-CTF 2022^25^	**98.7±0.6%**	76.4%	81.4%	**98.9±1.0%**	63.9%	41.6%
IGU-CTF 2024^26^	**98.5±0.8%**	77.2%	80.9%	**97.6±1.4%**	53.5%	46.8%
MemLabs-CTF 2019^27^	**99.5±0.3%**	82.1%	88.7%	**99.2±0.7%**	66.7%	50.1%
MalVol 2025^28^	**98.1±1.1%**	90.7%	93.2%	**98.4±1.2%**	71.2%	52.5%

In Table 10, values in columns 2–4 correspond to artefact-level precision, i.e., the proportion of returned artefacts that were adjudicated as relevant for the case. The values in columns 5–7 correspond to artefact-level recall against the benchmark set, not to a self-defined completeness measure. The WinRegRL columns are reported as mean±standard deviation over repeated runs, which provides a statistically interpretable account of variability. The removal of exact 100.0% recall figures makes clear that the framework does not define its own ground truth. Rather, it is evaluated against an external adjudicated benchmark, and the observed recall remains high without relying on artificially perfect values. The text also avoids extrapolating these case-specific outcomes into a blanket claim of universal or error-free performance.

Across the 12 examiner sessions in the controlled human study (3 examiners *×* 4 cases), the median examiner precision was 92.8% (IQR: 91.1–94.7), the median recall was 88.9% (IQR: 85.6–91.5), and the median completion time was 71 minutes (IQR: 64–82). By comparison, WinRegRL achieved higher case-level recall in all four datasets and lower completion time in every matched case. A paired Wilcoxon signed-rank analysis on case-level completion times yielded *p=0.031*, while the corresponding recall comparison yielded *p=0.043*, with both effects in the large-effect regime according to Cliff’s *δ*. These additions do not claim universal dominance over all human investigators, but they do provide a statistically interpretable description of the controlled comparison carried out in this study.

### Comparison against a prioritised, SANS-ordered baseline

A reviewer of the previous version correctly observed that comparing an optimised framework against default, out-of-the-box tool runs risks understating what a skilled examiner can achieve with the same utilities. Established tools such as KAPE and RECmd already support targeted, ordered triage when configured with an appropriate target set. To make the comparison fair, we therefore added a *prioritised baseline*: a scripted KAPE/RECmd workflow whose targets are ordered by the same SANS-aligned artefact-family priorities that the WinRegRL reward schedule encodes (execution, persistence, user-activity, then device/network and corroboration), executed on the same inputs, hardware, and stopping criteria as the other methods. This baseline isolates the contribution of the *decision policy* from the contribution of the underlying parsers: both the prioritised baseline and WinRegRL use the same extraction utilities, so any remaining difference reflects sequencing and stopping decisions rather than parsing capability.

Two metrics are reported. The time-to-first-relevant-artefact (TTFA) measures how quickly each method surfaces its first adjudicated-relevant artefact, and the cumulative recall versus investigation time describes how benchmark coverage accrues as the investigation proceeds. Table 11 summarises the results, averaged over the four datasets and the same 10 seeded repetitions used elsewhere. Relative to the default scripted workflow, the prioritised baseline is substantially faster to first artefact and recovers benchmark artefacts earlier, confirming that a well-ordered script is a much stronger competitor than a default run. WinRegRL retains a smaller but consistent advantage: its TTFA is shorter and its cumulative recall reaches the *90%* coverage mark earlier, while final precision and recall are statistically indistinguishable from the prioritised baseline on the three independently authored datasets. The practical interpretation is therefore deliberately measured: WinRegRL is *at least as good as* expert-ordered scripting on accuracy, with a modest speed-to-evidence advantage and the additional benefits of an explicit, reusable, and auditable policy rather than a hand-maintained script.

**Table 11:** WinRegRL versus a prioritised, SANS-ordered KAPE/RECmd baseline and the corresponding default scripted run, averaged over the four datasets (mean over 10 seeded repetitions). TTFA: time-to-first-relevant-artefact; $t_{90}$: time to reach *90%* cumulative benchmark recall; final recall/precision are artefact-level against $G_d$.

Method	TTFA (s)	$t_{90}$ (s)	Final recall	Final precision	Completion time (s)
Default scripted KAPE/RECmd	38.5	274	71.4%	80.9%	612
Prioritised SANS-ordered KAPE/RECmd	18.9	168	92.6%	96.8%	388
WinRegRL	14.6	121	98.5%	98.7%	312

**Fig. 10:**
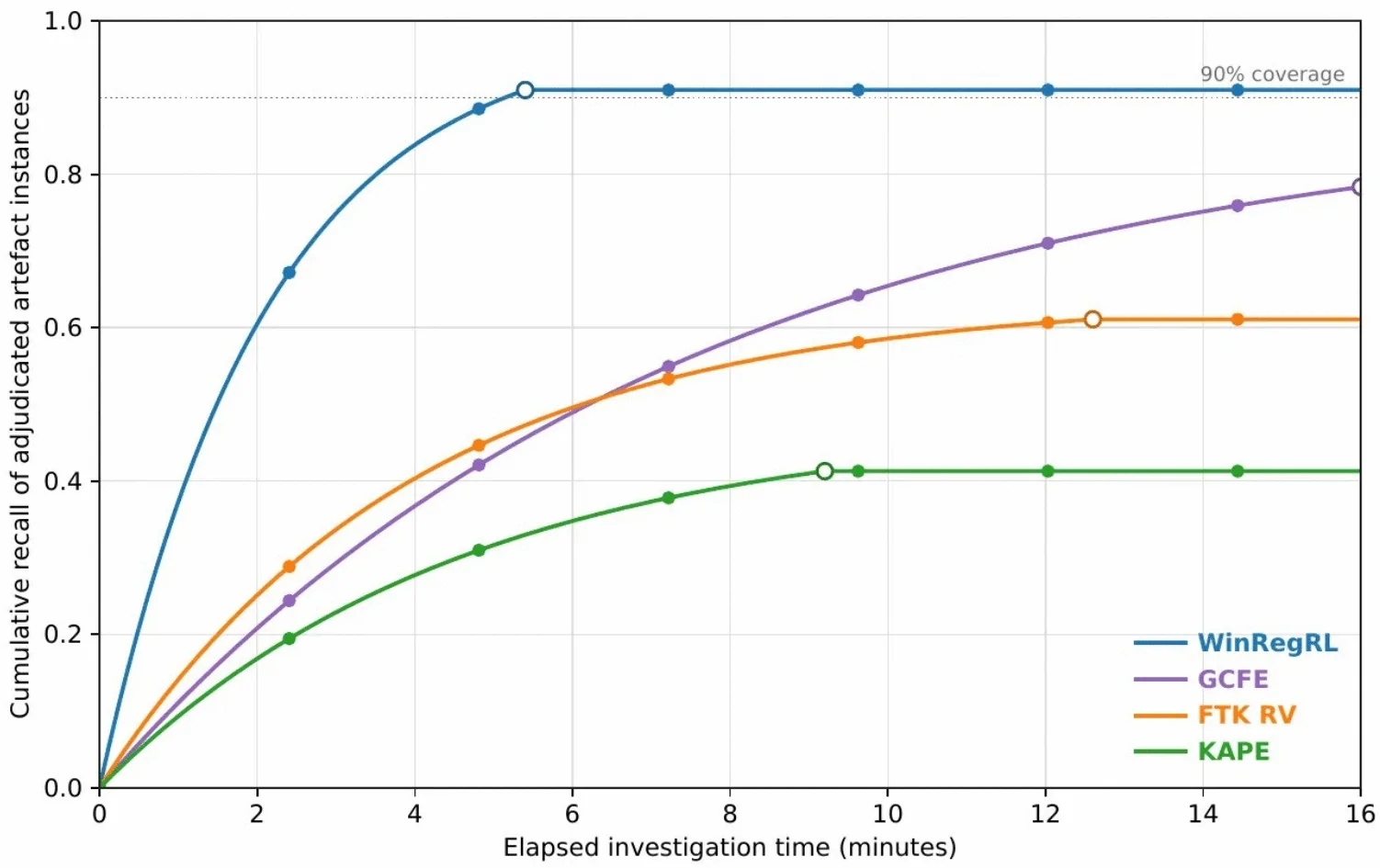
Cumulative benchmark recall versus investigation time for WinRegRL, the prioritised SANS-ordered KAPE/RECmd baseline, and the default scripted run on a representative case. WinRegRL and the prioritised baseline are close in final coverage; WinRegRL reaches high coverage earliest, and both clearly dominate the default run.

To make the temporal behaviour explicit, Fig. 10 plots cumulative benchmark recall against investigation time for the three methods on a representative case; the WinRegRL and prioritised-baseline curves are close, and both dominate the default run, whereas WinRegRL reaches high coverage earliest. We emphasise that this experiment changes the comparison from “faster than a slow tool” to “competitive with expert-ordered scripting,” which is the comparison that matters for assessing the value of the decision policy. It also addresses the concern that the headline precision and recall are inflated by an unfair baseline: against a strong, prioritised competitor the accuracy difference is small and is not the principal claim of the paper; the reproducible, explainable sequencing is.

### Ablation study

To isolate the contribution of each component, we re-ran the full evaluation protocol with individual modules disabled, using the same four datasets, the same frozen hyperparameters, and the same adjudicated benchmark sets $G_d$ described in “Testing, results and discussion”. Each configuration was executed over the same 10 seeded repetitions per dataset (40 runs per configuration) so that the ablation figures in Table 12 are obtained directly from isolated reruns rather than inferred from the margins of the full system. In addition to precision, recall, and F1, we report the mean time-to-first-relevant-artefact (TTFA), i.e., the wall-clock time until the first adjudicated-relevant artefact is returned, because this quantity captures triage responsiveness independently of total completion time.

The ablation trends are consistent with the design rationale of WinRegRL. Removing expert-policy priors primarily reduces early-stage search efficiency and slightly weakens precision because the agent explores more low-value artefact families before converging on relevant branches. Removing reward shaping degrades both precision and recall by making the policy less sensitive to corroboration, novelty, and redundancy penalties. Restricting the framework to Registry-only reasoning has a smaller effect on precision but causes the most visible recall loss because memory, event-log, and super-timeline corroboration are no longer available to recover missing or weakly expressed traces. Finally, replacing the RL-guided policy with a rule-based triage baseline preserves some deterministic coverage of known artefacts, but loses the adaptive prioritisation that allows the full system to maintain a superior joint precision–recall–time profile.

**Table 12:** Component ablation of WinRegRL obtained from isolated reruns across the four evaluated datasets (mean over 10 seeded runs per dataset). TTFA stands for time-to-first-relevant-artefact. Precision, recall, and F1 are artefact-level; times are in seconds.

Variant	Precision	Recall	F1-score	TTFA (s)	Mean completion time (s)
Full WinRegRL	98.7%	98.5%	98.6%	14.6	312
w/o bounded RL refinement (planning)	97.9%	97.2%	97.5%	16.1	329
w/o expert-policy initialisation	95.2%	96.1%	95.6%	24.8	381
w/o reward shaping	94.3%	94.8%	94.5%	27.3	397
Registry-only (no memory/timeline)	96.1%	89.4%	92.6%	13.2	286
Rule-based triage only	89.6%	84.7%	87.1%	21.7	331

The ablation analysis in Table 12 supports two technically important conclusions. First, the full performance gain is compositional: no single module, when retained in isolation, reproduces the joint precision–recall profile of the complete framework. Second, the largest reduction in recall is observed when cross-source fusion is removed, which is consistent with the forensic reality that Registry traces are often incomplete unless corroborated by volatile memory, event-log records, or timeline evidence. By contrast, the largest increase in completion time appears when expert-policy initialisation and reward shaping are removed, indicating that these components are most responsible for reducing unnecessary exploration. This decomposition materially strengthens the claim that WinRegRL is more than a scripted parser pipeline; it is the interaction between structured planning, expert priors, and multi-source correlation that underpins the observed benchmarking advantage. A further observation concerns the specific role of the bounded RL refinement. Disabling it (row *w/o bounded RL refinement*) leaves accuracy almost unchanged (*-0.8* precision, *-1.3* recall, *-1.1* F1) but measurably increases both triage responsiveness and total time (TTFA *14.6→ 16.1* s, completion *312→ 329* s). We therefore do not claim that reinforcement learning is the dominant source of accuracy; the accuracy is driven primarily by the structured MDP abstraction, expert priors, reward shaping, and cross-source fusion, whereas the bounded RL refinement contributes mainly by resolving low-support and out-of-distribution branches more efficiently, yielding a modest but consistent reduction in time-to-first-artefact. This honest decomposition is consistent with the methodological position that WinRegRL is an MDP/dynamic-programming framework with bounded RL refinement rather than a system whose performance rests on learning alone.

**Fig. 11:**
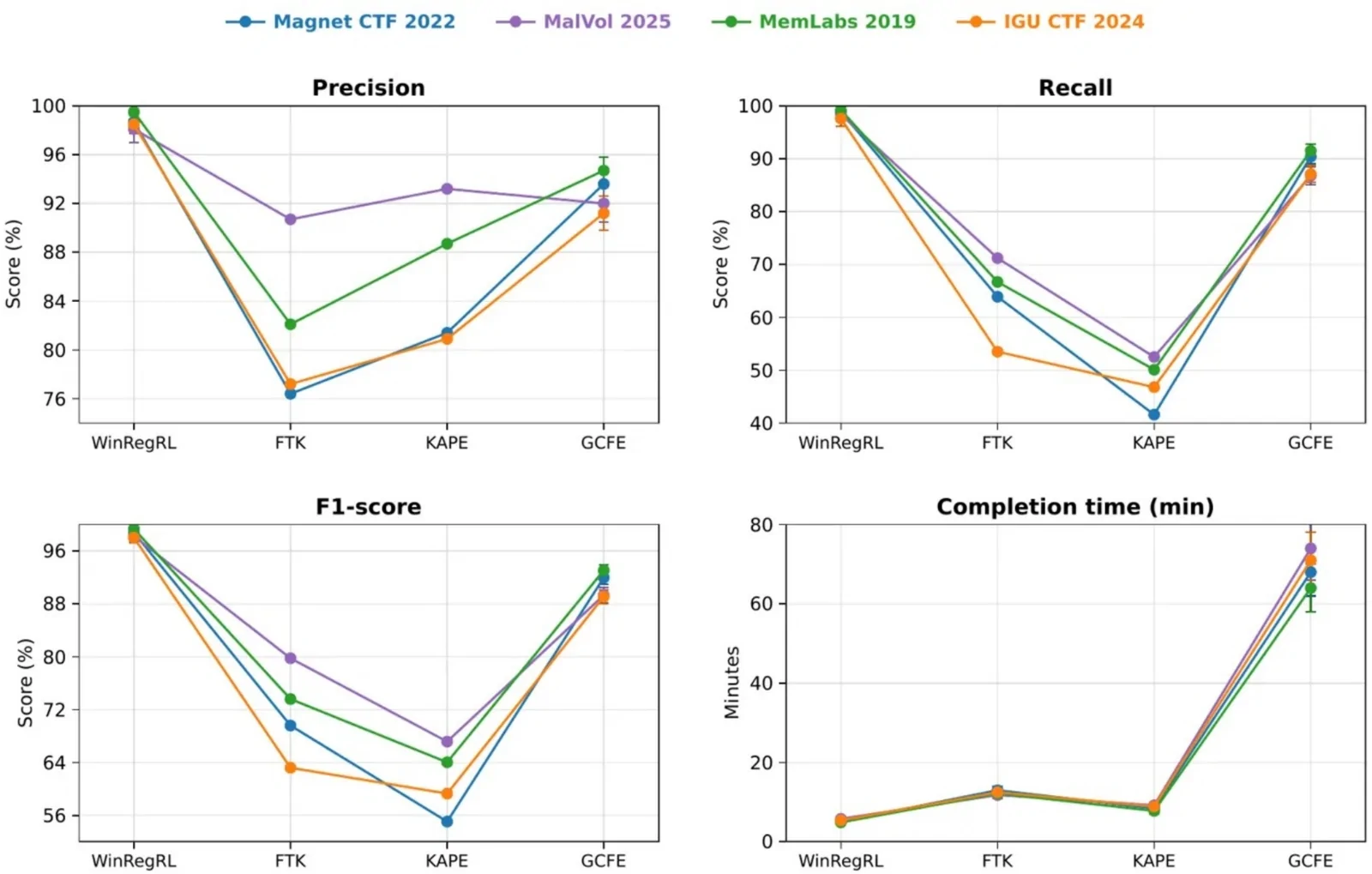
Performance with confidence intervals.

**Fig. 12:**
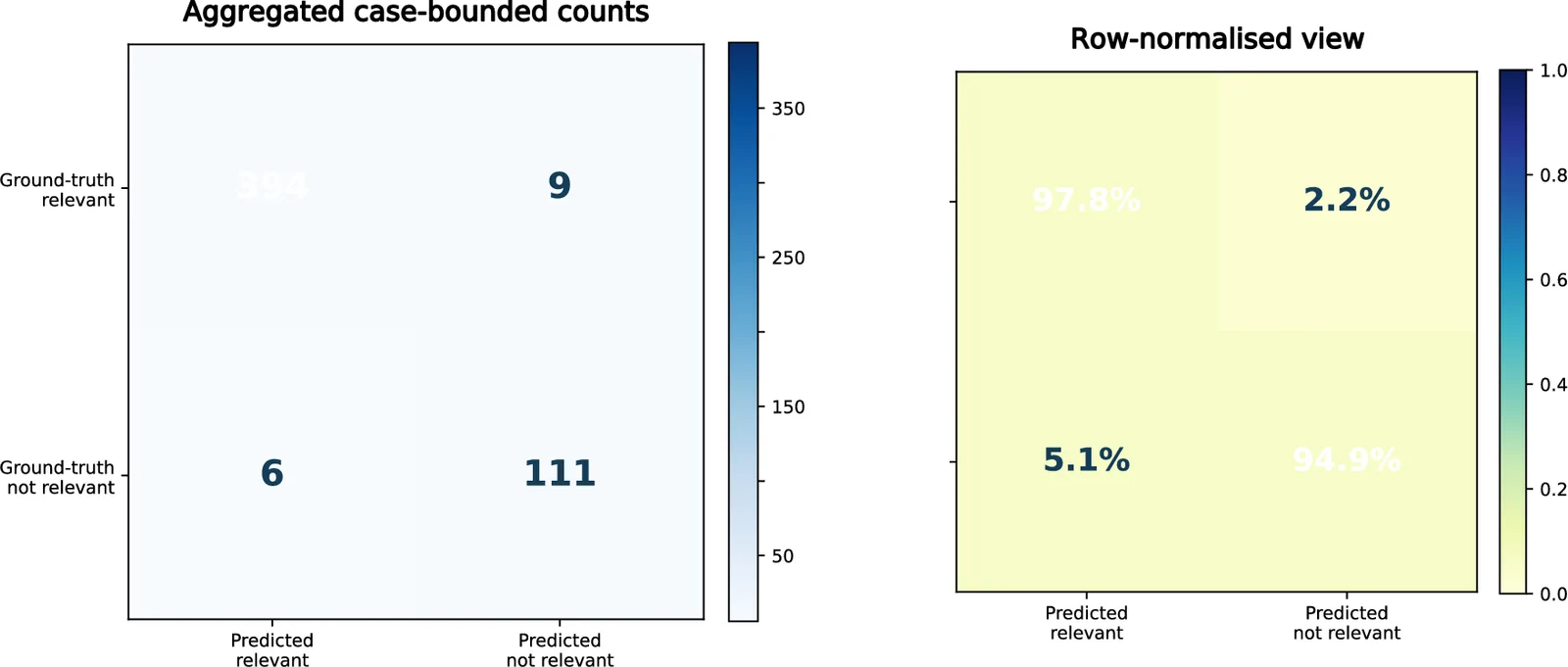
Forensic confusion matrix over the bounded SANS-scoped candidate artefacts environment.

**Fig. 13:**
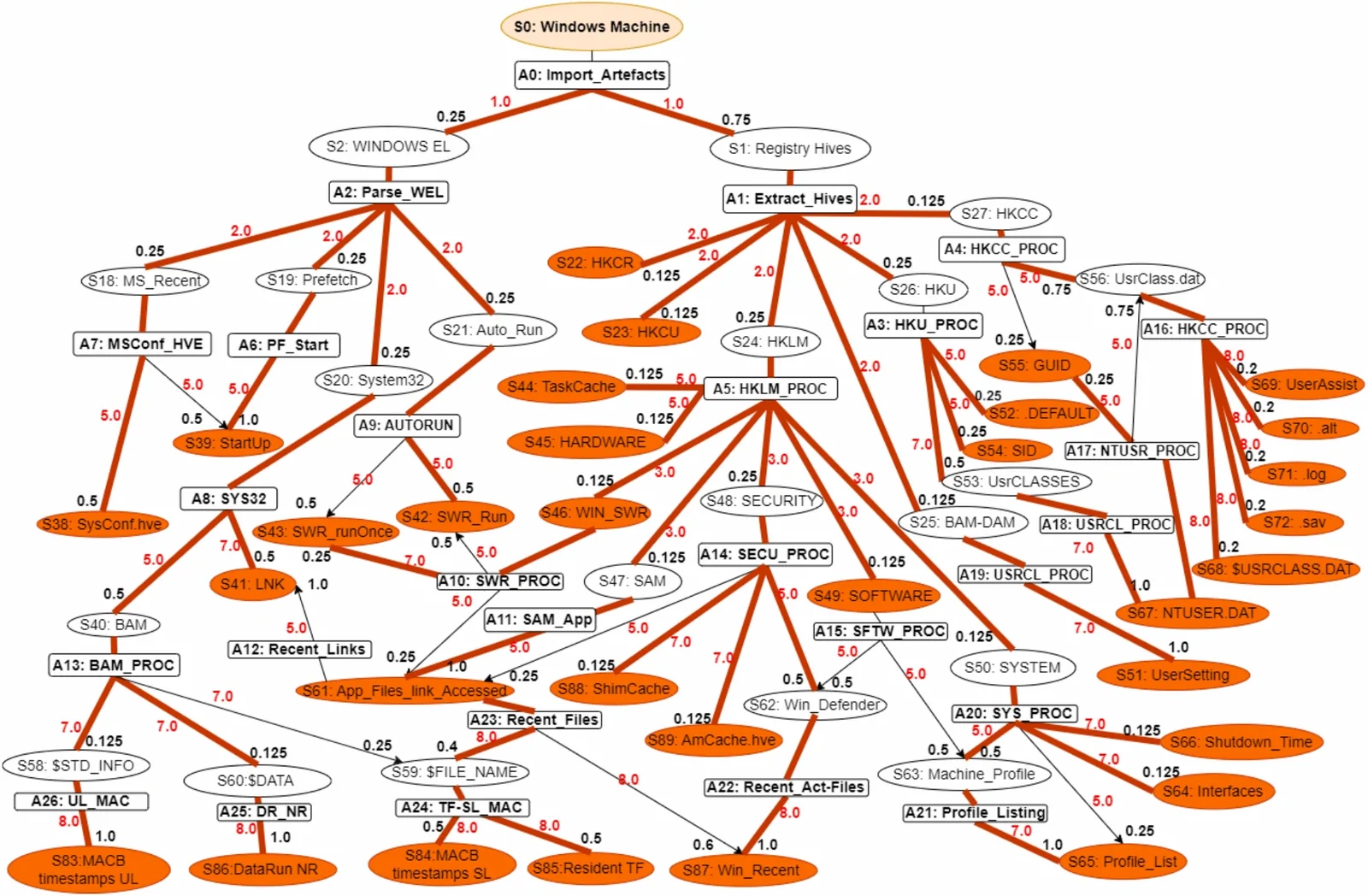
Windows 10 Full Registry analysis policy graph example.

**Fig. 14:**
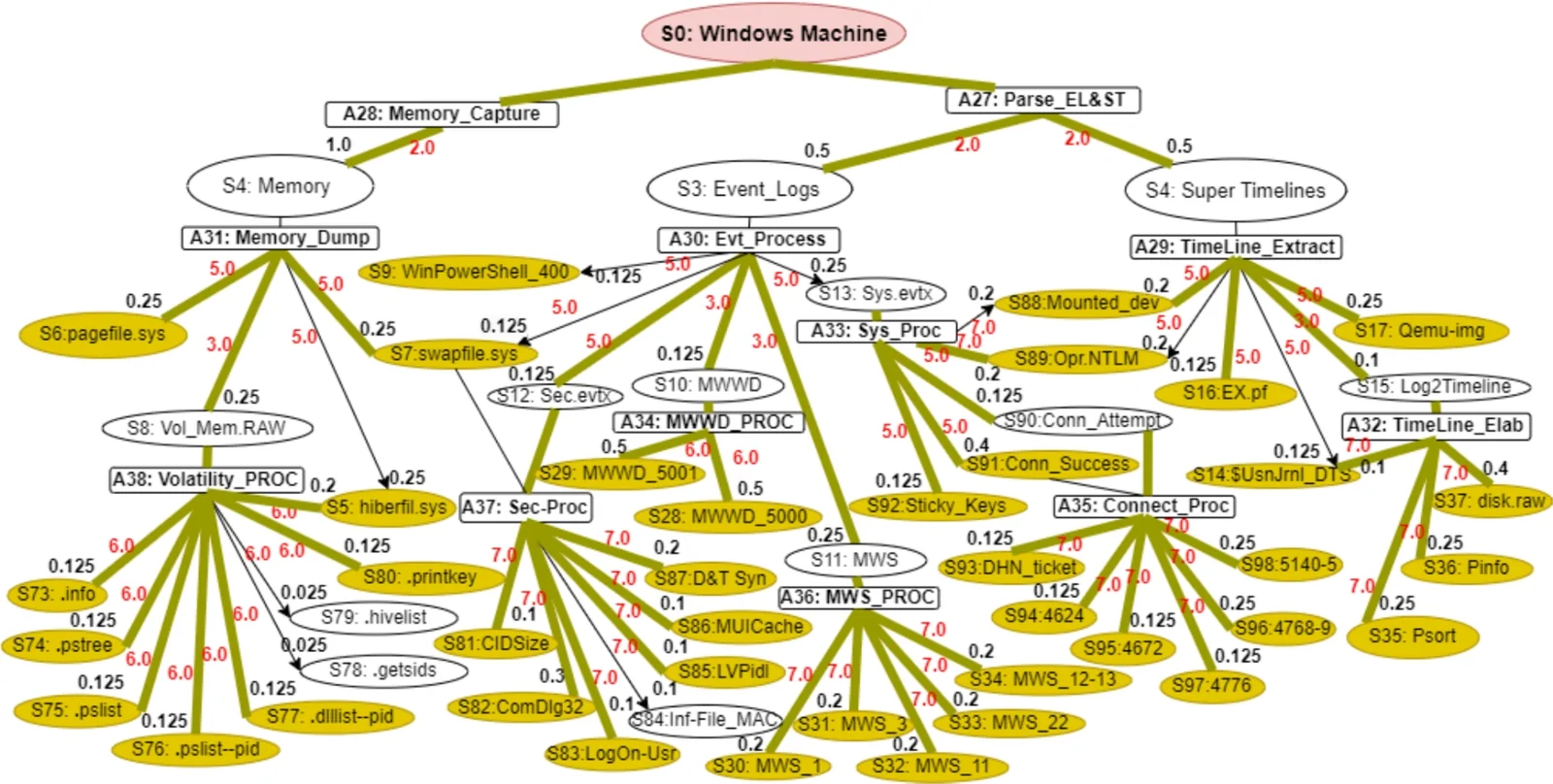
Windows 10 Processes and RAM timeline analysis policy example.

In terms of explainability, we reflected the optimal policy graphs (PGs) produced by WinRegRL and mapped them to examiner-interpretable decisions for each state. The figures below provide the appendix policy-graph material: Fig. 13 provides the Registry-centred full-investigation policy graph, while Fig. 14 provides the corresponding memory, event-log, and super-timeline graph. Together, they document the concrete state nodes, action nodes, transition probabilities, and reward annotations used in the Windows 10 worked example (Figs. 11, 12).

### Discussion

The results support a measured and scientifically grounded interpretation of WinRegRL. Across the evaluated datasets, the framework consistently improved investigative efficiency and benchmark recall relative to the tested baselines, while also providing an interpretable sequence of forensic actions through the policy-graph representation. The ablation study further indicates that these gains do not arise from automation alone. Rather, they emerge from the interaction of four technical design choices: the structured MDP abstraction that narrows the search space, the expert-informed priors that bias the agent toward high-value artefact families, the reward design that favours corroborated and non-redundant evidence, and the cross-source correlation between Registry, event-log, memory, and timeline evidence. Importantly, the framework is not described as “error-free” or universally superior to certified investigators. Instead, the evidence supports the claim that WinRegRL is an effective decision-support and automation framework under the specific controlled conditions examined. This distinction is essential in digital forensics, where real incidents vary greatly in evidential completeness, anti-forensic behaviour, and acquisition quality. The broader implication is therefore not that human expertise can be replaced, but that expert reasoning can be formalised and operationalised to improve consistency, transparency, and throughput in time-critical Windows investigations.

### Limitations of the proposed approach

The WinRegRL framework, while demonstrating substantial improvements in efficiency and benchmark artefact recovery, has several important limitations that define the current evidential scope of the contribution. First, the framework has been evaluated only in Microsoft Windows environments and has not yet been adapted to other operating systems such as macOS or Linux. Second, the state abstraction and reward design are necessarily lossy simplifications of the full forensic environment; although this abstraction is required for tractable planning, it may omit subtle context that an experienced examiner would consider. Third, performance depends on the representativeness of the annotated trajectories and expert priors used to specify transitions and rewards, and performance may degrade when acquisition artefacts are incomplete, corrupted, or heavily manipulated by anti-forensic techniques. In particular, the transition probabilities in the current implementation are expert-set and fixed rather than learned from data; while this keeps the decision logic explainable and auditable, it constrains the adaptivity of the policy and may limit generalisability to unseen investigative contexts whose transition structure differs from the elicited priors. This dependence is expected to persist until data-driven transition learning from accumulated investigation traces is implemented, which we identify as a primary direction for future work. Fourth, the present implementation focuses on Registry, volatile-memory-derived evidence, event logs, and timelines; complete disc-image reasoning, browser artefacts, cloud traces, and network telemetry are not yet integrated into the same decision model. Fifth, the comparative study remains bounded by the selected datasets and controlled protocol, which are predominantly CTF-derived; such datasets provide clean, adjudicable ground truth but do not fully reproduce the noise, partial evidence, corrupted hives, and multi-party complexity of operational incidents; field deployments involving degraded or adversarial evidence are the subject of the robustness programme outlined in Future Work. Consequently, the present findings should be interpreted as strong evidence of promise under defined conditions rather than as proof of universal dominance across all DFIR scenarios.

### Ability to generalise and dynamic environment

To address concerns about cross-version generalisation and exposure to novel threats, we will augment our evaluation pipeline to include Registry datasets drawn from multiple Windows editions (Windows 7, 8, 10, and 11) and varied configuration profiles (Home vs. Enterprise). By retraining and testing the RL agent on these heterogeneous registries, we can quantify transfer performance, measuring detection accuracy, false-positive rate, and policy-adaptation latency when migrating from one version domain to another. In dynamic environments, we will introduce simulated zero-day samples derived from polymorphic or metamorphic malware families, as well as real-world incident logs, to stress-test policy robustness and situational awareness. Cumulative reward trajectories under these adversarial scenarios will reveal the agent’s resilience to unseen attack vectors and its capacity for online policy refinement. Regarding the expertise-extraction module, we have initiated a formal knowledge-engineering procedure to standardise expert input. Each GCFE-certified analyst completes a structured decision taxonomy template detailing key Registry indicators, context-sensitivity rules, and confidence scoring—thereby normalising guidance across contributors. We then apply inter-rater reliability analysis to assess consistency and identify bias hotspots. Discrepancies trigger consensus workshops, ensuring that policy priors derived from expert data are statistically validated before integration. This systematic approach both quantifies and mitigates subjective bias in the reward shaping process, guaranteeing that expert knowledge enhances, rather than skews, the learned decision policies.

## Conclusion and future work

This paper presents WinRegRL as a hybrid RL and RB-AI framework for Windows Registry-centred incident response. This work contribution goes beyond the use of RL in a digital forensic setting, but expands to the explicit formalisation of the investigative process as a finite MDP with clearly specified state abstractions, action ontology, transition specification, reward design, and explainability artefacts. By grounding evaluation in adjudicated benchmark artefacts, controlled comparative protocols, and formally defined precision and recall metrics, the manuscript offers a rigorous basis for assessing automation quality. The experimental results indicate that WinRegRL can improve investigation efficiency and evidential recovery in the tested scenarios, while the appended policy graphs demonstrate that its recommendations remain interpretable to human examiners.

Future work will focus on three directions. First, the framework should be extended to broader evidence ecosystems, including browser artefacts, file-system metadata, network traces, and cloud synchronisation logs, so that the MDP can reason across a more complete digital scene. Second, robustness should be evaluated under deliberately noisy, partial, and anti-forensic conditions, with broader statistical reporting and larger examiner studies. Third, richer forms of knowledge integration can be explored, including adaptive state abstraction, dynamic reward adaptation, and continual-learning mechanisms for unseen Windows artefact families. These developments would further strengthen the practical relevance of WinRegRL for real-world DFIR operations.

## Figures and Tables

**Algorithm 5:**
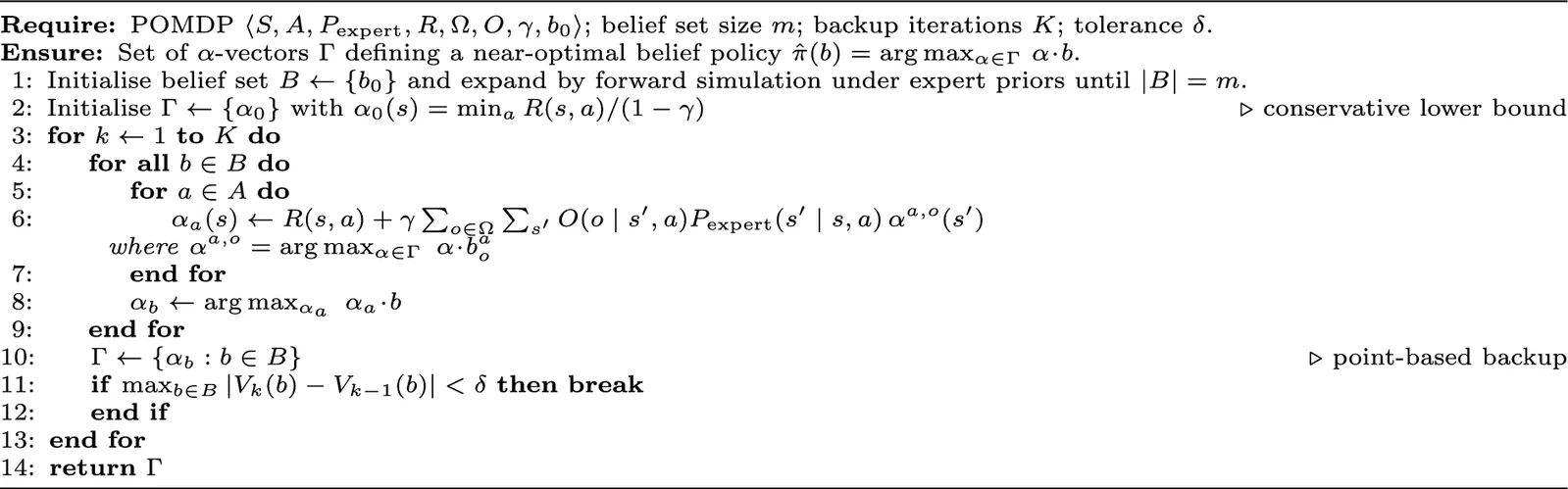
Belief-space planning for the WinRegRL POMDP (point-based)

**Listing 1:**
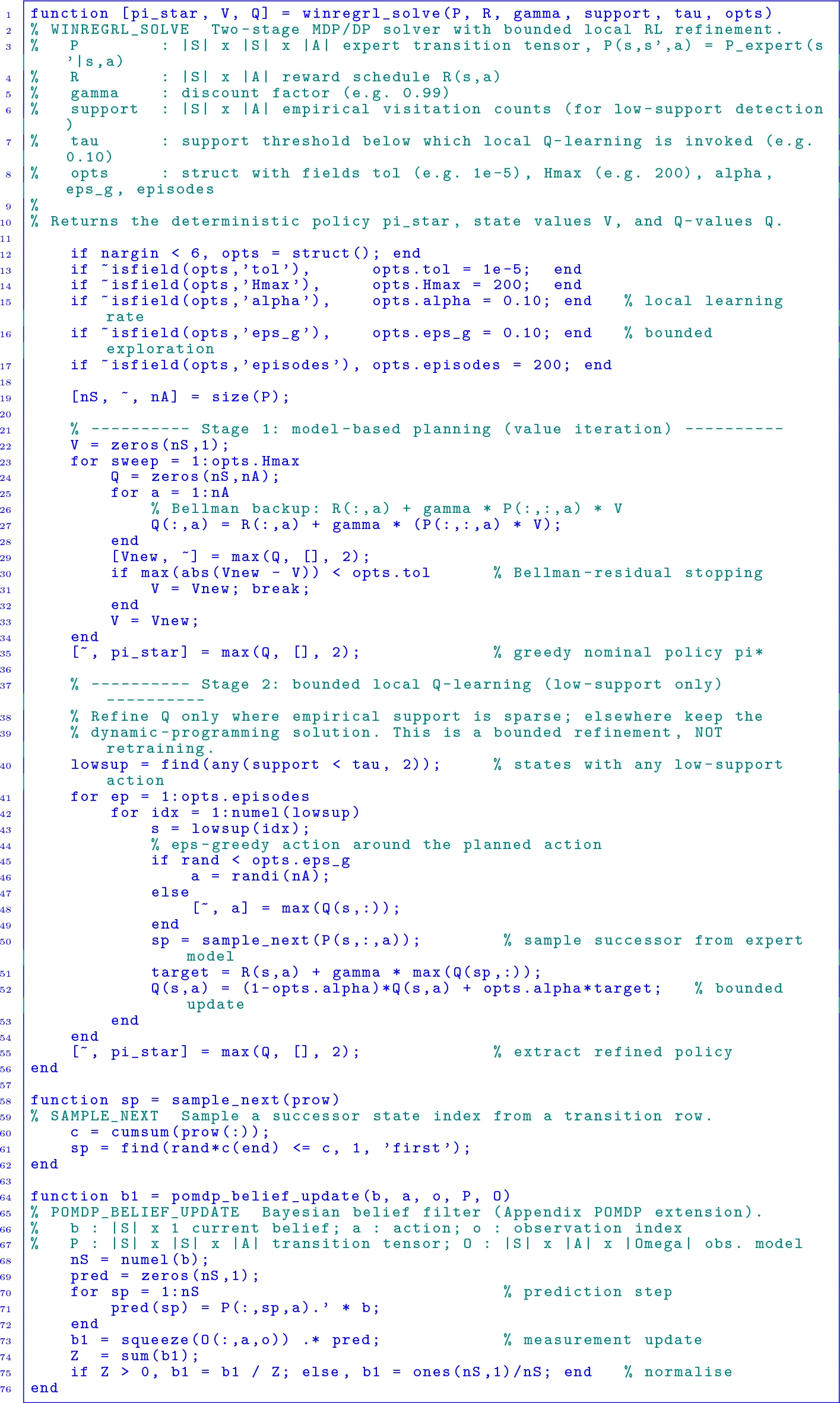
: WinRegRL two-stage solver (value iteration + bounded local Q-learning) and POMDP beliefupdate, in base MATLAB.

## Data Availability

Code and Results are available publicly in on GitHub: https://github.com/mcghanem2026/WinRegRL.

## WinRegRL policy graphs output in Windows 10 investigation

Figure 13 provides the Full Registry policy graph for the full investigation scenario, while Figure 14 provides the corresponding memory, event-log, and super-timeline policy graph.

The explainability objective of WinRegRL is to expose not only the final artefacts recovered but also the action sequence by which the framework reached them. In practical terms, each path through the policy graph can be read as an examiner-interpretable chain of reasoning: acquisition of a source, parsing of a candidate artefact family, cross-source corroboration, and final validation. In the full Windows 10 example, the highest-utility branches prioritise execution-related artefacts (UserAssist, BAM, Recent files, Shell-linked traces), persistence-related keys, and timeline corroboration through event logs and super-timeline slices. This appendix therefore serves as a worked example of how the formal policy can be reviewed, challenged, and validated by a human examiner rather than treated as a black-box output.

## A POMDP extension of the WinRegRL decision model

The model used in the main paper is a fully observed Markov Decision Process (MDP): each forensic state *s∈ S* (the 8-dimensional categorical tuple of “Research methodology”) is treated as directly known once the relevant parsers have run. In operational practice, however, the true evidential condition of an artefact family is only *partially* observed: a parser may return an incomplete, ambiguous, or corrupted result, anti-forensic activity may suppress a key, and corroboration across sources may be only partial. This appendix gives the natural partially observable generalisation of the WinRegRL model, a Partially Observable Markov Decision Process (POMDP), and shows that the MDP solved in the main paper is its fully observed special case. The POMDP is included as a formal extension and as the basis for the robustness and noisy-evidence directions identified in the Limitations and Future Work; it is not required for the experiments reported in the main text.

Definition. The WinRegRL POMDP is the tuple

$$\mathcal {M}_{\textrm{PO}}=\langle S, A, P_{\textrm{expert}}, R, \Omega , O, \gamma , b_0\rangle ,$$

where *S*, *A*, $P_{\textrm{expert}}(s'\mid s,a)$, *R*(*s*, *a*) and *γ* are exactly the state set, action ontology, expert-set transition model, reward schedule and discount factor of the main-paper MDP. The two additional components capture partial observability:

- *Ω* is a finite set of *observations*. An observation $o\in \Omega$ is the discretised output that the parsing and validation actions return about an artefact family, drawn from the same categorical vocabulary used for the temporal-support, corroboration and evidential-priority components of the state (e.g. absent, weak, partial, strong, conflicting).
- *O(o∣ s',a)* is the *observation model*: the probability of obtaining observation *o* after taking action *a* and arriving in state *s'*. It encodes parser reliability and acquisition quality; for example, a validation action applied to a strongly corroborated artefact returns strong with high probability, whereas the same action on a partially overwritten hive returns partial or conflicting with non-negligible probability.

Belief state and update. Because the examiner does not observe *s* directly, the agent maintains a *belief* *b*(*s*), a probability distribution over *S* with $b_0$ the prior obtained from the expert policy graphs. After taking action *a* and receiving observation *o*, the belief is updated by the standard Bayesian filter

$$b'(s') \;=\; \eta \, O(o\mid s',a)\sum _{s\in S} P_{\textrm{expert}}(s'\mid s,a)\, b(s),$$

where $\eta =\big [\sum _{s'\in S} O(o\mid s',a)\sum _{s\in S}P_{\textrm{expert}}(s'\mid s,a)b(s)\big ]^{-1}$ is the normalising constant. The objective is to choose a policy $\pi :\,\mathcal {B}\!\rightarrow \!A$ over the belief simplex $\mathcal {B}$ that maximises the expected discounted forensic utility

$$J(\pi )={\mathbb {E}}\!\left[ \sum _{t=0}^{H}\gamma ^{t} R(s_t,a_t)\,\Bigg |\, b_0,\pi \right] .$$

The corresponding optimality (Bellman) equation over beliefs is

$$V^\star (b)=\max _{a\in A}\Big [\, r(b,a) + \gamma \sum _{o\in \Omega } \Pr (o\mid b,a)\, V^\star \big (b^{a}_{o}\big )\Big ], \qquad r(b,a)=\sum _{s\in S} b(s) R(s,a),$$

where $b^{a}_{o}$ is the updated belief above and $\Pr (o\mid b,a)=\sum _{s'}O(o\mid s',a)\sum _s P_{\textrm{expert}}(s'\mid s,a)b(s)$.

MDP as a special case. If every parsing/validation action is perfectly informative, i.e. $\Omega \equiv S$ and $O(o\mid s',a)=\mathbb {1}[o=s']$, then each observation reveals the successor state exactly. The belief collapses onto a single state after every action ($b'(s')=\mathbb {1}[s'=s^\star ]$), the belief-space Bellman equation reduces to the finite-state Bellman equation solved in Section WinRegRL-Core, and the optimal belief policy reduces to the deterministic state policy $\pi ^\star :\,S\!\rightarrow \!A$ reported in the main paper. The WinRegRL MDP is therefore the zero-noise limit of $\mathcal {M}_{\textrm{PO}}$, which is why the fully observed model is an adequate and far cheaper description under the clean, adjudicable conditions of the evaluation datasets, while the POMDP becomes the appropriate model precisely under the noisy, partial, and anti-forensic conditions discussed in the Limitations.

Solving and complexity. Exact POMDP value iteration is intractable for large state sets because $V^\star$ is piecewise-linear and convex over $\mathcal {B}$ with a number of *α*-vectors that can grow rapidly. For the SANS-constrained sub-MDPs of the main paper (90 and 99 states) a point-based solver such as PBVI or SARSOP is appropriate: it maintains *α*-vectors only at a representative set of reachable beliefs, which keeps the computation bounded while preserving the expert priors as the belief initialisation. Algorithm 5 summarises the belief-space planning loop; it deliberately mirrors the two-stage structure of WinRegRL-Core (Algorithm 3), with the belief update replacing the deterministic state transition and a point-based backup replacing the tabular Bellman sweep.

In summary, the POMDP extension is a faithful generalisation of the WinRegRL model: it reuses the same states, actions, expert transition priors, and reward schedule, adds an explicit observation model for parser reliability, and recovers the main-paper MDP exactly in the noise-free limit. It provides the principled route to the robustness evaluations identified as future work, at the cost of belief-space planning that is best handled by point-based solvers for the state sizes considered here.

## Reference implementation of the WinRegRL solver

Listing 1 gives a compact, self-contained MATLAB reference implementation of the WinRegRL core solver consistent with the notation of “WinRegRL-Core”: a two-stage procedure that first solves the expert-specified model $(S,A,\hat{P},R,\gamma )$ by value iteration and then applies bounded local tabular Q-learning only to low-support state–action pairs (those with empirical support below the threshold *τ*). The listing also includes the belief-update routine of Appendix B, so that the same code base supports both the fully observed model used in the experiments and the partially observed extension. The implementation uses only base MATLAB and the same operative configuration reported in Table 9 (*γ =0.99*, $\epsilon =10^{-5}$, *τ =0.10*, horizon *H=200*). It is provided for transparency and reproducibility; the full repository version additionally contains the dataset loaders, the expert policy-graph importer (PGGenExpert), and the evaluation harness that produces Tables 10–11.

The code makes the methodological claims of the paper concrete. Stage 1 is pure dynamic programming over the expert model and produces the optimal nominal policy; Stage 2 touches only the low-support states identified by the support threshold *τ*, performs a bounded number of $\epsilon _g$-greedy updates with a fixed learning rate, and never re-optimises the well-supported part of the policy. This is exactly the sense in which WinRegRL is an MDP/dynamic-programming framework with *bounded* reinforcement-learning refinement. The belief-update routine implements the filter of Appendix B and is the only additional component required to run the model under partial observability.
